# Nanostructured Materials in Glucose Biosensing: From Fundamentals to Smart Healthcare Applications

**DOI:** 10.3390/bios15100658

**Published:** 2025-10-02

**Authors:** Rajaram Rajamohan, Seho Sun

**Affiliations:** School of Chemical Engineering, Yeungnam University, Gyeongsan 38541, Republic of Korea

**Keywords:** glucose biosensors, nanotechnology, various sensory materials, recent strategy, future focus

## Abstract

The rapid development of nanotechnology has significantly transformed the design and performance of glucose biosensors, leading to enhanced sensitivity, selectivity, and real-time monitoring capabilities. This review highlights recent advances in glucose-sensing platforms facilitated by nanomaterials, including metal and metal oxide nanoparticles, carbon-based nanostructures, two-dimensional materials, and metal–organic frameworks (MOFs). The integration of these nanoscale materials into electrochemical, optical, and wearable biosensors has addressed longstanding challenges associated with enzyme stability, detection limits, and invasiveness. Special emphasis is placed on non-enzymatic glucose sensors, flexible and wearable devices, and hybrid nanocomposite systems. The multifunctional properties of nanomaterials, such as large surface area, excellent conductivity, and biocompatibility, have enabled the development of next-generation sensors for clinical, point-of-care, and personal healthcare applications. The review also discusses emerging trends such as biodegradable nanosensors, AI-integrated platforms, and smart textiles, which are poised to drive the future of glucose monitoring toward more sustainable and personalized healthcare solutions.

## 1. Introduction

### 1.1. Diabetes Patients Globally

**Figure 1:**
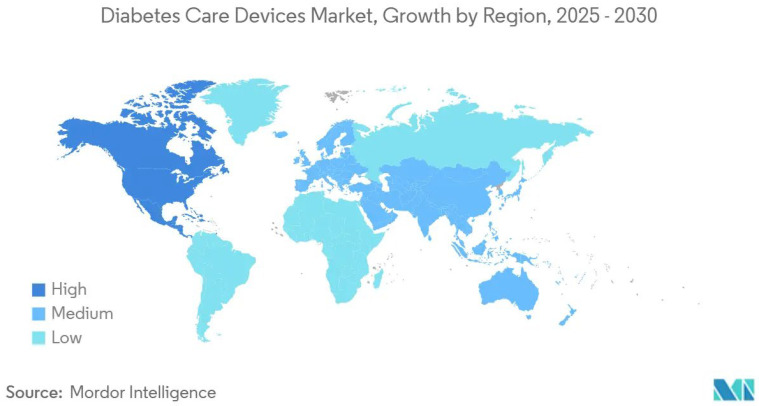
Worldwide diabetes data. Reprinted from [2] as it is the IDF atlas.

In 2024, North America held 40.7% of the global market, supported by Medicare reimbursement and a high density of trained endocrinologists. The CDC reported 29.7 million diagnosed and 8.7 million undiagnosed diabetes cases, with the latter offering growth potential through pharmacy-linked screening [1]. Yet, rising cost pressures from payers may compress premium continuous glucose monitoring (CGM) prices, steering adoption toward value-based models. The Asia–Pacific region is set for the fastest growth (8.2% CAGR through 2030), fueled by urbanization and the world’s largest diabetes population (Figure 1), representing over 60% of global cases according to the International Diabetes Federation (IDF) diabetes Atlas [2]. The global diabetes care devices market is projected to expand significantly between 2025 and 2030, with the highest growth observed in North America and Australia, driven by advanced healthcare infrastructure and early adoption of innovative technologies. Europe, Asia–Pacific, and South America are expected to exhibit medium growth, supported by rising diabetes incidence and increasing awareness of glucose monitoring solutions. Conversely, Africa and parts of the Middle East show relatively low growth, reflecting healthcare access challenges and cost limitations. Its digitally engaged population may favor smartphone-integrated CGMs over earlier models, with companies offering localized AI-driven coaching poised for outsized market gains. In Europe, growth remains steady, sustained by universal healthcare and aging demographics. The EMA’s stricter trial demands slow commercialization, but the Abbott–Dexcom patent settlement has removed legal uncertainty. The patent settlement between Abbott and Dexcom marked a major milestone in the CGM market, while stringent FDA regulations continue to pose challenges that can delay the commercialization of medical devices [3]. With clearer procurement timelines, hospitals are likely to negotiate bulk-purchase discounts, compressing prices while boosting adoption.

Diabetes is a growing global health challenge, affecting individuals, families, and nations [4]. According to the IDF Diabetes Atlas 2025, 11.1% of adults (1 in 9) aged 20–79 live with diabetes, with over 40% undiagnosed. By 2050, the number is projected to rise to 1 in 8 adults ( ≈ 853 million), a 46% increase [2]. There are two main types of diabetes. Type 1 diabetes results from the autoimmune destruction of pancreatic cells, causing absolute insulin deficiency. It is most common in children and adolescents [5] but can occur at any age [6]. Type 2, accounting for over 90% of cases, arises from insulin resistance and impaired insulin production. Its rise is driven by urbanization, ageing, reduced physical activity, and increasing obesity [7,8,9]. Preventive measures for type 2 diabetes, along with early diagnosis and proper care for all types, can reduce complications and improve quality of life.

### 1.2. Clinical Urgency and Monitoring

Diabetes poses urgent clinical risks. Acute complications of uncontrolled blood sugar include hypoglycemia, hyperglycemia, ketoacidosis, and coma, while chronic complications involve retinopathy, nephropathy, neuropathy, and cardiovascular disease [10]. Diagnosis and monitoring rely on several tools. HbA1c testing reflects average glucose over ~3 months but lacks real-time data [11]. Self-monitoring of blood glucose (SMBG), recommended ≥ 3 times daily for intensive insulin therapy, uses finger-prick meters to guide treatment [11]. CGM represents a major advance, tracking glucose day and night via a subcutaneous sensor, transmitter, and receiver or smartphone app. CGM reduces hypoglycemia risk, lowers HbA1c, and provides real-time insights [12]. However, each method has drawbacks: SMBG involves pain, infection risk, and sampling errors [13], while CGM may require calibration, face sensor drift, and occasional finger-prick confirmation [14].

### 1.3. The Critical Need for Advanced Glucose Biosensors

The growing prevalence of diabetes and related metabolic disorders underscores the urgent need for reliable glucose monitoring tools. Real-time glycemic management [15,16,17,18] provides a comprehensive framework for diabetes care, enabling early detection and timely correction of dysglycemia. Continuous and accurate monitoring supports individualized insulin dosing strategies, thereby improving patient safety and clinical outcomes.

Furthermore, the development of non-invasive, wearable, and user-friendly glucose biosensors enhances patient adherence, comfort, and overall quality of life. Such devices reduce the burden of frequent finger-prick testing and encourage consistent self-management. Importantly, the utility of glucose biosensors extends beyond conventional diabetes care, offering applications in gestational diabetes monitoring, early identification of prediabetes, and assessment of metabolic health in broader populations.

### 1.4. Introduction to Glucose Biosensing

Glucose biosensors are vital in diabetes management, originating from the first enzyme electrode [19]. Over time, they have advanced in sensitivity, selectivity, and accessibility, evolving from simple prototypes to miniaturized, commercially available devices. Modern biosensors can detect glucose in blood, interstitial fluid, and alternative biofluids like sweat and tears, enabling real-time, continuous, and often non-invasive monitoring for improved diabetes care [20].

### 1.5. Generational Advancements

**Figure 2:**
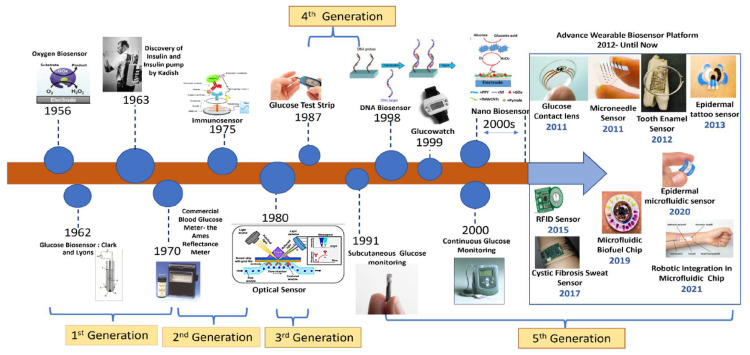
The different milestones achieved in the field of biosensors in terms of year. Reproduced [21] with the permission of the Creative Commons Attribution 4.0 International License (http://creativecommons.org/licenses/by/4.0/). Copyright 2022, MDPI.

The evolution of glucose biosensors is divided into four generations based on detection principles (Figure 2) [21].

The development of glucose biosensors has progressed through five distinct generations, each driven by advances in science and technology to overcome the limitations of earlier systems. The first generation (1962–1970s) began with the pioneering glucose biosensor based on the Clark oxygen electrode (1962) and the discovery of immobilized enzymes (1963) by Leland C. Clark and Champ Lyons. These early devices relied on the natural oxygen consumption of glucose oxidase reactions but were hindered by oxygen dependence and interference from electroactive species. The second generation (1970s–1980s) addressed these limitations through mediator-based biosensors, enabling electron transfer independent of oxygen. This era marked the commercialization of glucose biosensors (1975) for self-monitoring of blood glucose and the emergence of optical sensors in the 1980s, which laid the foundation for portable, home-use glucose meters. The third generation (1980s–1990s) was characterized by direct electron transfer between the enzyme and electrode, leading to greater accuracy and reliability. The introduction of glucose test strips in 1987 revolutionized convenience and portability, while the early 1990s saw the concept of subcutaneous glucose monitoring, paving the way for minimally invasive devices. The fourth generation (2000s–2010s) witnessed a transformative integration of nanotechnology and DNA-based sensing strategies. Key milestones included the introduction of DNA sensors and devices like the GlucoWatch (1998–1999), followed by continuous glucose monitoring (CGM) systems in the early 2000s. With the expansion of nanobiosensors, detection sensitivity and device miniaturization have significantly improved. From 2011 onwards, a new wave of advanced wearable platforms emerged, such as glucose contact lenses (2011), microneedle sensors (2011), tooth enamel tattoo sensors (2012), epidermal tattoo sensors (2013), RFID sensors (2015), cystic fibrosis sweat sensors (2017), microfluidic biofuel chips (2018), epidermal microfluidic sensors (2020), and robotic integration in microfluidic chips (2021). The fifth generation of glucose biosensors (present–future) represents a paradigm shift toward smart, non-invasive, and fully integrated biosensing platforms. Current advancements emphasize the development of wearable, implantable, and lab-on-chip systems that provide continuous, real-time monitoring while minimizing patient discomfort. These next-generation devices are designed to support personalized medicine by enabling tailored treatment strategies and seamless wireless data transmission. Moreover, the integration of artificial intelligence (AI), the Internet of Things (IoT), and robotics into biosensing platforms is paving the way for predictive healthcare, where real-time data analytics not only monitor glucose fluctuations but also anticipate dysglycemic events, thus offering proactive and precision-driven disease management.

Third-generation sensors aim for direct electron transfer between GOx and the electrode, enabled by nanostructured materials such as graphene, carbon nanotubes, and metallic NPs. These materials enhance conductivity, catalytic activity, and stability, advancing reliable real-time glucose monitoring (Table 1).

The fourth generation of glucose biosensors, or non-enzymatic sensors, eliminates enzyme dependence by using the direct electro-oxidation of glucose on nanostructured electrodes with high intrinsic electrocatalytic activity [33]. Transition metals (Pt, Au, Ni, Cu, Co) and their oxides, hydroxides, or alloys act as active sites to drive electron transfer during glucose oxidation [34]. Sensitivity and durability have been enhanced through advanced nanomaterials such as metal nanoparticles, metal–organic frameworks, doped carbon nanostructures, graphene, and carbon nanotubes. However, challenges remain, including poor selectivity from electroactive interferents, reduced stability under physiological conditions, and the need for alkaline media. Ongoing efforts in understanding nanomaterial catalysis and designing 3D architectures hold promise for next-generation, enzyme-mimicking non-enzymatic biosensors that may overcome these barriers and enable reliable real-time glucose monitoring.

### 1.6. Sensor Formats & Media

Electrochemical methods, particularly amperometric and voltammetric techniques, remain the cornerstone of glucose biosensing due to their high sensitivity, rapid response, and suitability for point-of-care use. Recent innovations have moved beyond traditional blood testing toward wearable and implantable formats for non-invasive or minimally invasive sampling. These systems analyze alternative biofluids such as sweat, interstitial fluid, and tears, enabling CGM with real-time feedback, reduced discomfort, and improved long-term adherence. Glucose biosensors operate through enzymatic or electrochemical principles, typically employing electrodes (working, reference, and counter) and enzymes such as glucose oxidase (GOx) or glucose dehydrogenase (GDH). Detection media include whole blood, interstitial fluid, and optimized solutions like phosphate buffer to ensure reliable performance (Table 2).

### 1.7. Enzyme and Non-Enzyme Glucose Biosensors

Enzymatic glucose biosensors rely on specific enzymes, most notably GOx and glucose dehydrogenase (GDH), to catalyze the oxidation of glucose. Their high substrate specificity ensures excellent selectivity and sensitivity, making them the dominant choice in commercial glucose monitoring systems. However, their performance can be limited by environmental factors such as temperature, pH, and humidity, as well as by enzyme instability and oxygen dependence, which may reduce long-term reliability (Table 3). In contrast, non-enzymatic glucose biosensors utilize catalytic nanomaterials with strong intrinsic electrocatalytic activity to directly oxidize glucose. Materials such as transition metals (Ni, Cu, Co, Pt, Au) and their oxides or hydroxides (e.g., NiO, CuO, Co_3_O_4_) have been widely investigated. These systems offer advantages, including enhanced stability, lower cost, and independence from oxygen levels, while also tolerating harsher physiological and environmental conditions. To further improve sensitivity, durability, and biocompatibility, recent approaches incorporate nanostructured composites such as carbon nanotubes, graphene, and metal–organic frameworks, which increase surface area, electron transfer efficiency, and catalytic activity.

## 2. Recent Advances in Glucose Biosensors via Nanotechnology

**Figure 3:**
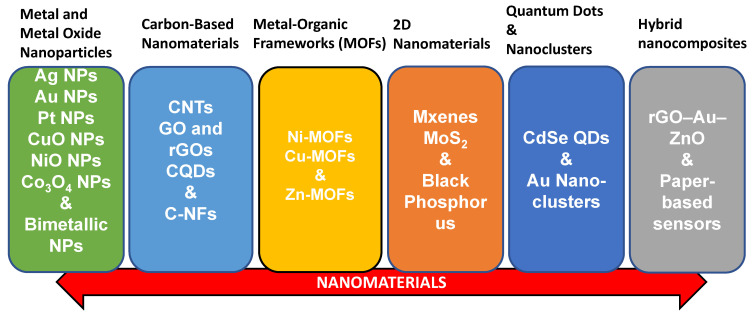
Various types of nanomaterials for an efficient glucose biosensor.

Nanotechnology has revolutionized glucose sensing, offering innovative strategies to enhance sensitivity, lower detection limits, shorten response times, and enable the development of miniaturized devices suitable for point-of-care and personalized healthcare. The integration of nanomaterials has been particularly influential, as their unique physicochemical properties, such as high surface area, tunable morphology, superior conductivity, and strong catalytic activity, facilitate efficient electron transfer while improving biocompatibility. Metal nanoparticles (e.g., gold, silver, platinum, nickel, copper) are widely employed to amplify electrochemical signals and boost catalytic activity. Carbon-based nanostructures, including carbon nanotubes (CNTs), graphene, and graphene oxide, contribute high conductivity, chemical stability, and versatile scaffolds for enzyme or mediator immobilization. More recently, two-dimensional (2D) materials such as transition metal dichalcogenides (TMDs) and MXenes have expanded opportunities for next-generation biosensors, leveraging their layered architectures, high electron mobility, and tunable surface chemistry. Collectively, these nanotechnology-driven innovations not only elevate the analytical performance of glucose biosensors but also drive the development of flexible, wearable, and non-invasive monitoring systems, aligning with the increasing demand for continuous glucose monitoring and personalized diabetes management. This review focuses on the above-mentioned important categories as follows (Figure 3).

### 2.1. Metal and Metal Oxide Nanoparticles

Nanostructured metals are key to advancing glucose biosensors by enhancing electrocatalysis and increasing surface area for glucose oxidation, thereby improving sensitivity and detection efficiency [35]. Noble metal nanoparticles (Pt, Au, Ag) offer excellent conductivity, stability, and enzyme immobilization, boosting electron transfer in enzymatic sensors. In contrast, transition metal oxides (CuO, NiO, Co_3_O_4_) are widely used in non-enzymatic sensors, where their intrinsic redox activity enables direct glucose oxidation. Recently, bimetallic nanoparticles (e.g., Pt–Ni, Cu–Zn) have gained attention for their synergistic effects, providing higher catalytic activity, sensitivity, and durability, making them strong candidates for next-generation glucose monitoring (Table 4).

#### 2.1.1. Ag NPs

Several studies have demonstrated the use of Ag NPs to enhance glucose sensor performance. Tang et al. [36] employed Ag NPs to improve direct electron transfer between the electrode and GOx, immobilizing GOx in silver sol and cross-linking with polyvinyl butyral in glutaraldehyde on a platinum electrode. This modification increased electron transfer rates, resulting in a steady-state response three times higher than GOx alone, as Ag NPs acted as conductive pathways between enzyme prosthetic groups and the electrode surface. Manno et al. [37] leveraged the localized surface plasmon resonance (LSPR) of Ag NPs for optical glucose sensing. Ag NPs capped with polyvinyl alcohol showed a selective LSPR response only to glucose, while other sugars and buffers caused no change in absorbance, confirming specificity. Xia et al. [38] developed a colorimetric SPR-based glucose sensor using homogeneous Ag nanoplates. The sensor enabled real-time glucose detection in serum at micromolar concentrations. Increasing glucose levels induced a blue shift in the SPR band and a visible color change (blue → purple → mauve). Linear correlations between SPR peak shifts (Δλ) and glucose concentrations were observed in the 0.2–100 μM range, with minimal interference from other sugars or amino acids. Ensafi et al. [39] reported an electrochemical sensor using Ag NPs decorated on ligands bound to silica supports (SiO_2_-pro-NH-cyanuric-NH_2_ and SiO_2_-pro-NH_2_), facilitating efficient glucose detection. Li et al. [40] introduced an ATR fiber-optic glucose sensor enhanced with Ag NPs grown on the surface. Using a U-shaped fiber with a 0.25 cm radius and a CO_2_ laser light source, the Ag NP-modified ATR sensor achieved approximately threefold higher sensitivity and resolution compared to conventional ATR sensors, measuring glucose absorption at multiple infrared wavelengths (1037–1081 cm^−1^).

Ag NPs significantly improve glucose sensor performance by enhancing electron transfer, catalytic activity, and optical responses. Enzymatic sensors benefit from Ag NPs as conductive bridges, increasing sensitivity, while LSPR- and SPR-based optical sensors offer highly selective, real-time, and colorimetric glucose detection. Ag NP-decorated ligands and ATR fiber-optic sensors further extend their utility, providing high-resolution, continuous, and minimally invasive glucose monitoring.

#### 2.1.2. Au NPs

Gold nanoparticles (Au NPs) significantly enhance glucose biosensors by increasing electrode surface area, improving catalytic activity, and facilitating efficient electron transfer, which allows higher GOx enzyme loading, greater sensitivity, and lower detection limits. Chen et al. [41] developed a chitosan–AuNPs (CHI–Au NPs) nanocomposite on a glassy carbon electrode (GCE) Via a simple electrodeposition method. The sensor showed a detection limit (LOD) of 3.70 × 10^−4^ M and linearity from 4.00 × 10^−4^ M to 1.07 × 10^−2^ M for glucose. Au NP-anchored nitrogen-doped graphene (Au NP/NG) nanohybrids offered excellent stability, reproducibility, and electrochemically active surface area, accelerating electron transfer between the electrode and glucose. Lee et al. [42] demonstrated that these nanohybrids provided rapid and sensitive electrochemical responses. Raj and Jena [43] designed a non-enzymatic glucose sensor using Au NPs self-assembled on a 3D silica network. In phosphate buffer (pH 9.2), Au NPs efficiently catalyzed glucose oxidation at a low potential (0.16 V) without redox mediators or enzymes. The catalytic activity increased with the AuNP surface area. Ekram et al. [44] reported an enzyme-free sensor based on graphite/SrPdO_3_ modified with Au NPs, enabling direct glucose oxidation. The sensor achieved a linear range of 100 µM to 6 mM and a LOD of 10.1 µM, outperforming Pt- and Pd-modified electrodes in sensitivity and electron transfer. Another study [45] fabricated a GOx-Au NP covalent sensor, which displayed a rapid electrocatalytic response, reaching 95% of steady-state current within 8 s at 0.3 V vs. SCE, along with high stability under continuous potential cycling.

A simple and efficient strategy has been developed to fabricate nanoporous gold (NPG) electrodes in NH_4_Cl solution for nonenzymatic glucose detection [46]. Incorporating NH_2_OH·HCl as an anodic depolarizer and reducing agent significantly increased the surface area, enhancing sensitivity. The NPG sensor showed a broad linear range (0.2–16 mM; 0.036–2.7 g L^−1^) at −0.4 V and achieved a high sensitivity of 3625 μA mM^−1^.cm^−2^ at 0.2 V in 0.1 M NaOH. Another study reported that NPG with smaller pore sizes yields higher sensitivity for nonenzymatic glucose detection [47]. The electrodes also suppressed interference from common organic species, highlighting their high selectivity and potential for biosensor applications. Furthermore, NPG catalysts with controlled surface crystallographic orientations have been applied for saccharide oxidation [48]. While fructose showed no response, glucose, galactose, and mannose produced clear oxidation signals. The onset potentials varied with the surface structure, with Au(100)-enhanced NPG showing ~100 mV lower oxidation potential than Au(111). A linear range of 10 μM–1.8 mM was achieved, comparable to enzymatic sensors. These findings demonstrate that tailoring the surface orientation of NPG electrodes can improve electrochemical sensor performance.

Au NPs enhance glucose biosensors by increasing electrode surface area, catalytic activity, and electron transfer efficiency. They are used in both enzymatic (GOx-loaded) and non-enzymatic sensors, achieving low detection limits, wide linear ranges, and rapid responses. Nanocomposites such as CHI–Au NPs and Au NP/NG improve stability and sensitivity, while self-assembled Au NPs and Au NP-modified electrodes enable mediator-free glucose oxidation. These advances demonstrate Au NPs’ versatility in both electrochemical and enzyme-based glucose sensing, supporting high-performance, real-time glucose detection in biological samples.

#### 2.1.3. Pt NPs

Pt NPs are extensively used in glucose biosensors due to their outstanding electrocatalytic properties, which enhance sensitivity, stability, and selectivity. Many studies have combined Pt NPs with various nanomaterials to improve sensor performance. D. Zhai et al. [49] developed a highly sensitive glucose enzyme sensor using Pt NPs-polyaniline (PA) hydrogel heterostructures. Pt NPs were uniformly loaded onto a 3D PA hydrogel matrix, combining the hydrogel’s conductivity and porosity with the catalytic activity of Pt NPs. The hydrogel facilitated high-density enzyme immobilization and substrate diffusion, while Pt NPs efficiently decomposed H_2_O_2_ generated during glucose oxidation. The sensor exhibited remarkable performance with a sensitivity of 96.1 μA·mM^−1^·cm^−2^, a fast response time of 3 s, a linear range of 0.01–8 mM, and a low LOD of 0.7 μM. Another study [50] incorporated GOx into a PA-montmorillonite (MMT)-Pt NP nanocomposite electrode. The sensor showed no interference from common biomolecules (glycine, urea, L-phenylalanine, ascorbic acid, L-tyrosine, D-galactose), maintained over 91.7% of its original signal after two months, and provided a wide linear range (10 μM–1.94 mM) with a low detection limit of 0.1 μM. It was successfully applied to glucose detection in human serum. A Pt-decorated hollow carbon sphere (Pt/HCSs) electrode was developed [51], immobilized with GOx using Nafion. Pt NPs (≈2.29 nm) were well dispersed on HCSs, catalyzing the oxidation and reduction of enzymatically liberated H_2_O_2_. The sensor exhibited high sensitivity (4.1 μA·mM^−1^), fast response (<3 s), a low LOD (1.8 μM), and a wide linear range (0.04–8.62 mM). It showed high stability, reproducibility, and minimal interference from ascorbic acid and uric acid, achieving good recovery in blood serum samples. Another approach [52] employed ultrafine Pt NPs (~3 nm) for a simple glucose sensor, achieving high sensitivity (137.7 μA·mM^−1^·cm^−2^), fast response (5 s), a low LOD (5 μM), and a linear range of 0.2–3.2 mM (R^2^ = 0.999). The effects of pH, applied potential, and common interferents on amperometric response were systematically evaluated.

Pt NPs enhance glucose biosensors by improving electrocatalytic activity, enzyme immobilization, and electron transfer, resulting in highly sensitive, fast, and stable sensors. Various strategies, including Pt NPs incorporated into polyaniline hydrogels, montmorillonite composites, hollow carbon spheres, and ultrafine dispersed NPs, have achieved low LOD (0.1–5 μM), wide linear ranges (0.01–8.62 mM), fast response times (<5 s), and minimal interference from common biomolecules.

#### 2.1.4. CuO NPs

**Figure 4:**
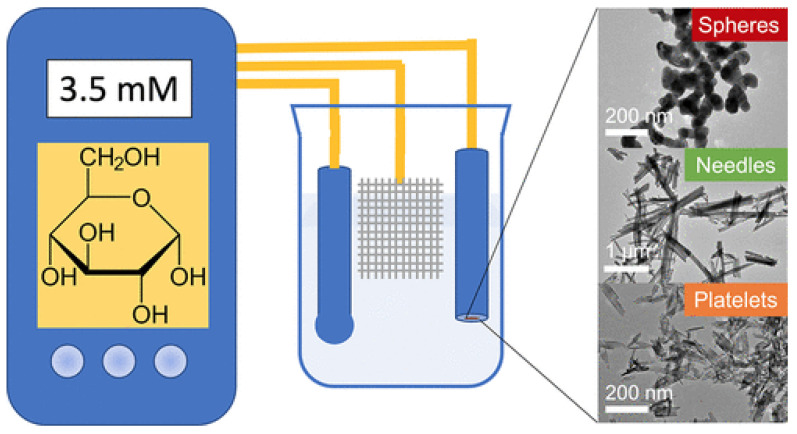
Scheme of synthesis of CuO NPs for glucose sensing. Reprinted with permission from American Chemical Society [53].

Copper oxide nanoparticles (CuO and Cu_2_O) have been widely explored for non-enzymatic glucose sensing due to their excellent electrocatalytic activity, low cost, and structural versatility. One study [53] investigated CuO particles with three morphologies: spheres, platelets, and needles by controlling synthesis parameters such as pH, precipitants, calcination, surfactant, and H_2_O_2_ addition (Figure 4). Morphology strongly influenced electrocatalytic performance, with needle-shaped CuO NPs providing the highest sensitivity (2.05 mA·mM^−1^·cm^−2^), wide linear range (0.05–5 mM), and superior long-term stability. Performance correlated primarily with grain size and capacitance. Another approach [54] fabricated nanostructured Cu_2_O/CuO electrodes with a columnar morphology using glancing angle deposition followed by thermal annealing. The Cu_2_O electrodes exhibited a high sensitivity of 1394 μA·cm^−2^·mM^−1^, a low LOD (0.052 μM), and two broad linear ranges (0.01–2 mM and 2–5 mM), with excellent reproducibility, selectivity, fast response, and accurate glucose measurements in blood serum. CuO NPs immobilized on mesoporous MCM-41 substrates [55] were developed Via solvothermal methods. The resulting CuO@MCM-41 sensor displayed a rapid 4 s response, high sensitivity (17.23 mA·cm^−2^·mM^−1^), a low LOD (16 nM), and a linear range of 83 μM–1.5 mM. Flexible glucose sensors were fabricated using laser-engraved graphene (LEG) electrodes decorated with CuO NPs [56]. These sensors exhibited high sensitivity (619.43 μA·mm^−1^·cm^−2^ for 0–3 mM, 462.96 μA·mm^−1^·cm^−2^ for 0–8 mM), excellent reproducibility, anti-interference ability, long-term stability, and mechanical flexibility, maintaining 82.4% of the initial current after 100 bending cycles. They also showed promising performance for wearable sweat sensing. CuO nanorods (NRs) were produced Via a two-step anodization method, with crystallite sizes of 17.8–49.6 nm and diameters of 10–60 nm depending on voltage [57]. These nanorods achieved high sensitivities (502.29–783.3 μA·mM^−1^·cm^−2^) and low LOD (0.35–0.5 μM). Finally, low-cost, partially flexible CuO microspheroid and urchin films were fabricated on laser-carbonized Nomex sheets for enzyme-free glucose sensing [58]. Cu microspheroids and CuO urchins exhibited high amperometric sensitivity (0.25–0.32 mA·cm^−2^·mM^−1^), chemical stability, and reproducibility, demonstrating potential for wearable and microfluidic point-of-care devices.

Copper oxide-based nanomaterials (CuO and Cu_2_O) demonstrate significant potential for non-enzymatic glucose sensors. Morphology, grain size, and nanostructure play critical roles in determining sensitivity, linear range, and stability. Needle-shaped CuO NPs, columnar Cu_2_O/CuO electrodes, mesoporous CuO@MCM-41, CuO-decorated graphene, and CuO nanorods all show high sensitivity, low LOD, rapid response, and selectivity, while flexible CuO films enable wearable and point-of-care applications. These studies highlight CuO’s versatility for both high-performance laboratory sensors and low-cost, flexible, or wearable glucose detection devices.

#### 2.1.5. NiO NPs

NiO NPs have been extensively explored for both enzymatic and non-enzymatic glucose sensing due to their excellent electrocatalytic properties, high stability, and tunable nanostructures. NiO NPs were synthesized Via an eco-friendly electro-exploding wire technique at 24, 36, and 48 V [59]. The NiO 48V sample exhibited the smallest particle size, rugged surface, and highest Ni^3+^ content, enhancing redox currents and glucose oxidation in alkaline media (0.1 M NaOH, pH 13). This sensor achieved a wide linear range (0.1–1 mM), high sensitivity (1202 μA·mM^−1^·cm^−2^), and a low LOD of 0.25 μM, with excellent stability, reproducibility, repeatability, and selectivity. Ultra-small NiO NPs prepared by calcination of nickel-p-phenylene diamine complexes [60] showed superior electrocatalytic activity compared to bulk NiO and bare electrodes. The NiO-modified GCE exhibited a linear range of 5 μM–2.49 mM, high sensitivity (0.310 μA·μM^−1^·cm^−2^), fast response ( < 5 s), low LOD (3.5 μM), and good selectivity and stability. A NiO NP-modified carbon paste electrode [61] functioned as an amperometric glucose sensor with high sensitivity (367 μA·mmol^−1^·L), a wide linear range (1.9 × 10^−3^–15 mmol·L^−1^), and a LOD of 0.11 μM. NiO-decorated graphene nanosheets (NiO/GNS), synthesized hydrothermally [62], showed enhanced catalytic activity due to graphene’s 2D structure, preventing NiO aggregation and facilitating electron transfer. The sensor had a linear glucose range of 5 μM–4.2 mM and a LOD of 5 μM. NiO/GNS was also used in biofuel cells, demonstrating high current density and stability. Nanostructured NiO thin films were deposited on F-doped SnO_2_ (FTO) Via RF magnetron sputtering [63]. GOx immobilization on these films enabled impedimetric glucose detection with a sensitivity of 4.45 kΩ/mM, a linear range of 0.2–4.0 mM, and a LOD of 24 μM. The high isoelectric point (~10.7) of NiO thin films favored enzyme adsorption. Bacteria-derived hollow cylinder NiO (HCNiO) enabled enzymeless glucose sensing [64]. In 0.05 M NaOH, the Ni^2+^/Ni^3+^ redox couple catalyzed glucose oxidation with high sensitivity (3978.9 μA·mM^−1^·cm^−2^) and a LOD of 0.9 μM, showcasing a green synthesis approach. Lastly, NiO NPs were electrodeposited onto SBA-15/MWCNT-modified carbon paste electrodes [65], producing a non-enzymatic sensor with a linear range of 0.06–400 μM and LOD of 0.023 μM. The sensor demonstrated rapid response, high sensitivity, and potential for point-of-care applications.

Nickel oxide nanoparticles (NiO NPs) are highly effective for glucose sensing due to their excellent electrocatalytic activity, tunable nanostructures, and high stability. Various strategies, including electro-exploding wire synthesis, calcination, hydrothermal NiO/graphene composites, thin films, hollow cylinder nanostructures, and SBA-15/MWCNT-supported NiO, have yielded sensors with high sensitivity (0.31–3978.9 μA·mM^−1^·cm^−2^), low detection limits (0.023–5 μM), wide linear ranges, fast responses ( < 5 s), and strong selectivity. These sensors are suitable for both enzymatic and non-enzymatic detection, with applications in biological fluids, biofuel cells, and wearable or point-of-care devices.

#### 2.1.6. Cobalt Oxide NPs

Cobalt oxide-based glucose sensors have been extensively developed for both enzymatic and non-enzymatic electrochemical detection due to their excellent electrocatalytic activity, stability, and tunable nanostructures. A non-enzymatic sensor was fabricated Via a simple chemical bath deposition of Co_2_(OH)_2_(CO_3_), which transformed into Co_3_O_4_ NPs upon electrochemical activation [66]. The sensor achieved ultrahigh sensitivity (33,245 µA·mM^−1^·cm^−2^), a linear range of 0–0.5 mM, and a LOD of 5 µM, maintaining stability over 12 months. An enzymatic glucose biosensor was developed by immobilizing GOx on a graphene/Co_3_O_4_ NPs composite [67]. This electrode exhibited fast electron transfer due to the biocompatibility of Co_3_O_4_ NPs and high conductivity of graphene, with a linear glucose range of 0.5–16.5 mM and sensitivity of 13.52 μA·mM^−1^·cm^−2^. The same composite also functioned as a non-enzymatic H_2_O_2_ sensor with <10 s response time. A ZnO/Co_3_O_4_/rGO nanocomposite prepared Via hydrothermal synthesis [68] served as a non-enzymatic glucose sensor with high catalytic activity, low working potential (0.55 V), and fast response (~3 s). It showed a wide linear range (0.015–10 mM), high sensitivity (1551.38 μA·mM^−1^·cm^−2^), and a LOD of 0.043 μM. Co_3_O_4_ NPs were also electrodeposited on GC electrodes Via potential cycling in tartrate-containing solutions [69], resulting in a non-enzymatic glucose sensor with linear ranges of 0.7–60 μM (batch) and 1.3–50 μM (flow), sensitivities of 2515.35 and 3240.25 μA·mM^−1^·cm^−2^, and LODs of 0.15 and 0.14 μM, respectively. A Ponagam seed shells-derived activated carbon/Co_3_O_4_ nanocomposite (PSAC/Co_3_O_4_) [70] functioned as a high-performance non-enzymatic glucose sensor with ultrahigh sensitivity (34.2 mA·mM^−1^·cm^−2^), very low LOD (21 nM), and long-term stability, also demonstrating supercapacitor performance. Finally, Co_3_O_4_ nanoflowers with a 3D hierarchical structure were synthesized and compared with spherical and nanorod shapes [71]. The nanoflowers showed superior electrocatalytic activity due to their high surface-to-volume ratio, achieving two linear ranges (5–60 μM and 0.2–3.0 mM) with sensitivities of 693.02 and 228.03 μA·mM^−1^·cm^−2^ and LODs of 0.04 and 0.14 μM, respectively, along with excellent selectivity.

Co_3_O_4_-based glucose sensors, both enzymatic and non-enzymatic, demonstrate high sensitivity, fast response, low LODs, wide linear ranges, and long-term stability. Strategies include electrochemical activation, composites with graphene or ZnO/rGO, electrodeposition, and shape-controlled nanostructures (spheres, nanorods, nanoflowers). Nanoflowers and activated carbon composites provide superior surface area and electrocatalytic behavior, making them highly effective for selective, sensitive, and stable glucose detection, with potential applications in point-of-care and wearable devices.

#### 2.1.7. Bimetallic Nanoparticles

Bimetallic NPs have proven to be highly effective in improving glucose biosensors due to the synergistic effect between two metals, which enhances sensitivity, selectivity, and stability. These nanomaterials often allow enzyme-free glucose detection, eliminating the limitations of enzymatic sensors such as short shelf life and susceptibility to environmental conditions. Common bimetallic combinations include gold-based, silver-based, and copper-based systems, with notable examples being Ni-Au, Au-Cu, and Cu-Ag. Acting as electrocatalysts, these NPs facilitate glucose oxidation and improve electrochemical responses, offering faster detection times and longer stability compared to traditional enzyme-based sensors.

Pt-Ni modified BDD electrodes were shown to provide a continuous and stable glucose response at neutral pH [72]. The sequence of electrochemical deposition significantly influenced morphology and electrocatalytic properties. The PtNi-BDD electrode displayed a 19% increase in initial signal after 200 cyclic voltammetry cycles (2400 s), compared with 44% and 40% for Ni/Pt-BDD and Pt-BDD, respectively. Sensitivity was measured at 110.4 μA cm^−2^ mM^−1^ over 2–12 mM glucose, approximately 6–15 times higher than single-metal electrodes or alternate-deposition configurations. Nickel was first deposited, followed by silver or copper to form Ag@Ni and Cu@Ni [73]. These high-surface-area bimetallic NPs exhibited superior electrocatalytic activity relative to Ni alone. When coated on titanium electrodes, they provided highly sensitive non-enzymatic glucose detection with low LOD. Fabricated in 0.4 M NaOH at 0.55 V (vs. Hg/HgO), these amorphous bimetallic NPs exhibited enhanced electrochemical oxidation efficiency compared with crystalline Ni–Co NPs [74]. The sensor achieved a high sensitivity of 2.455 mA mM^−1^ cm^−2^, a low LOD of 0.15 μM, and long-term stability. It was successfully applied for glucose measurement in beverages, showing recoveries from 95.6% to 105.6%. Pt-Ni alloy NPs dispersed on graphene-modified glassy carbon electrodes demonstrated excellent electrocatalytic performance [75]. Amperometric measurements showed a linear range of 0.5–15 mM, LOD of 16 μM, and high sensitivity of 24.03 μA mM^−1^ cm^−2^. The electrode exhibited good selectivity and durability, making it suitable for glucose detection in blood serum. A scalable synthesis method was developed to fabricate Cu-Ag bimetallic nanostructures Via galvanic replacement [76]. Optimized conditions (AgNO_3_ 5 mM, reaction time 30 s) produced nanostructures with enhanced electrocatalytic activity. The sensor displayed a fast response (~2 s) and high sensitivity (3802 μA cm^−2^ mM^−1^ for 0.01–3 mM and 712 μA cm^−2^ mM^−1^ for 3–8 mM) at an optimum potential of 0.65 V. The synergistic effect of Cu-Ag significantly improved performance compared to monometallic analogs.

Bimetallic NPs enhance glucose biosensing by combining two metals to exploit synergistic electrocatalytic effects. Key highlights include as following. Pt-Ni BDD electrodes: stable, continuous response, sensitivity 110.4 μA cm^−2^ mM^−1^ (2–12 mM). Ag@Ni and Cu@Ni: high-surface-area structures with superior electrocatalytic activity and low LOD for enzyme-free detection. Amorphous Ni–Co NPs on carbon cloth: high sensitivity (2.455 mA mM^−1^ cm^−2^), low LOD (0.15 μM), reliable for beverages. Pt-Ni alloy on graphene: linear range 0.5–15 mM, LOD 16 μM, selective and durable for blood serum glucose detection. Cu-Ag nanostructures: fast response (~2 s), dual linear ranges, enhanced activity due to synergistic effects. Hence, bimetallic NPs offer enzyme-free, highly sensitive, selective, and stable platforms for glucose detection, suitable for practical biomedical, food, and environmental applications.

### 2.2. Carbon-Based Nanomaterials

Carbon-based nanomaterials have emerged as key components in the development of high-performance glucose sensors due to their exceptional physicochemical and electrochemical properties. These materials, which include carbon nanotubes (CNTs), graphene, graphene oxide (GO), reduced graphene oxide (rGO), and carbon quantum dots (CQDs), are widely employed because of their high specific surface area, outstanding electrical conductivity, chemical stability, and intrinsic catalytic activity. The high surface area of these carbon nanomaterials provides a larger platform for enzyme immobilization or direct adsorption of glucose, allowing more active sites for glucose oxidation and improving overall sensor sensitivity. Their excellent electrical conductivity facilitates rapid electron transfer between the electrode surface and redox-active species, which is crucial for achieving a fast and efficient electrochemical response. Furthermore, the catalytic properties of certain carbon-based nanomaterials can promote the oxidation of glucose either directly (non-enzymatic sensors) or by enhancing enzymatic reactions, resulting in improved detection limits and response times.

Specifically, carbon nanotubes (CNTs) are widely recognized for their ability to enhance electron transfer kinetics due to their one-dimensional conductive network, which minimizes resistance and accelerates charge transfer at the electrode interface. CNTs also provide a mechanically robust and chemically stable support for enzyme immobilization, maintaining enzyme activity over extended periods and contributing to the long-term stability and reproducibility of the sensor. Similarly, graphene and its derivatives offer a two-dimensional conductive network with abundant active edges, enabling efficient electron transfer and strong interactions with biomolecules, while carbon quantum dots (CQDs) contribute fluorescence and additional active sites that can improve sensitivity and selectivity in both optical and electrochemical glucose sensors.

#### 2.2.1. Carbon Nanotubes (CNTs)

CNTs are widely used in glucose sensors due to their high surface area, excellent conductivity, and mechanical robustness, which make them ideal both as scaffolds for enzyme immobilization and as direct conductive materials for non-enzymatic sensing. A CNT microelectrode set (CNT µ-ES) was developed using highly densified CNT fibers (HD-CNTf) embedded in a polymer matrix with open-ended CNTs exposed at the interface [77]. The working electrode was modified with Cu NPs, the counter electrode was bare CNTf, and the quasi-reference electrode was Ag/AgCl-coated CNTf with Nafion™. Electrochemical tests in 0.1 M NaOH showed that the Cu NPs/HD-CNTf microsensor had an extremely low LOD (28 nM), high sensitivity (1942 nA·µM^−1^·cm^−2^), and minimal interference from physiological interferents. Fiber-like CuO-modified CNTs (CuO@CNTFs) were developed as flexible and wearable glucose sensors [78]. The CNT fibers’ unidimensional structure enhanced conductivity and mechanical strength, while electrodeposited CuO NPs provided electrocatalytic activity. These microelectrodes achieved high sensitivity (~3000 μA·mM^−1^·cm^2^), low LOD (1.4 μM), and a wide linear range (up to 13 mM). Low charge transfer resistance confirmed superior performance.

Functionalized CNTs combined with Co(OH)_2_ were used for a non-enzymatic glucose sensor, showing improved stability and reproducibility [79]. The sensor demonstrated good linearity over 50–700 μM glucose and a LOD of 43.2 μM, highlighting the feasibility of cost-effective CNT-based sensors. CNTs functionalized with specific groups also provide strong platforms for enzyme immobilization. For instance, a single-wall CNT field-effect transistor (FET) and CNT nanoelectrode ensembles (NEEs) were used for selective enzymatic glucose detection [80]. Covalent immobilization of glucose oxidase on CNT NEEs enabled detection Via catalytic reduction of hydrogen peroxide, showing high selectivity in the presence of common interferents without requiring membranes or electron mediators [81].

Hence, CNTs enhance glucose sensors through high surface area, conductivity, and mechanical stability. Non-enzymatic sensors using CuNPs or CuO@CNTFs offer low LODs, wide linear ranges, and high sensitivities. Functionalized CNTs improve enzyme immobilization, selectivity, and stability, enabling effective enzymatic glucose detection. CNT-based microelectrodes and NEEs allow interference-free detection without membranes or mediators, supporting flexible, wearable, and cost-effective glucose sensors.

**Figure 5:**
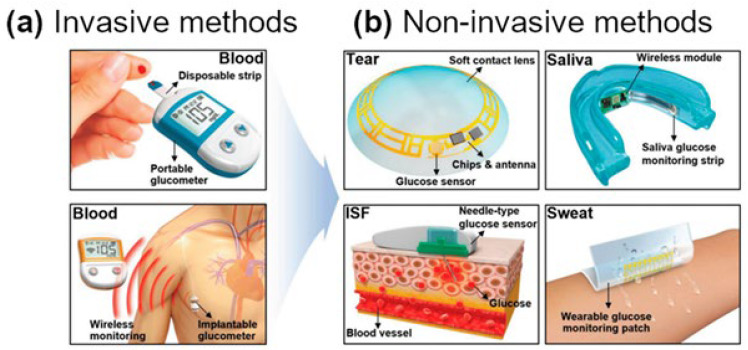
Evolution of glucose sensors using (**a**) invasive and (**b**) non-invasive methods [83]. Reproduced with permission of the Creative Commons Attribution 4.0 International License (http://creativecommons.org/licenses/by/4.0/). Copyright 2021, MDPI.

There is increasing interest in non-invasive glucose sensors [82], which can be implanted or applied to the skin to measure glucose in fluids such as sweat, saliva, tears, or interstitial fluid (Figure 5a, and 5b) [83]. GOx immobilized on CNTs provides a highly sensitive electrochemical platform for low-concentration glucose detection [84]. Upon contact, glucose is oxidized to gluconic acid, altering the sensor’s electrochemical properties. Kang, Park, and Ha demonstrated a CNT-based wearable sensor capable of detecting glucose down to 50 μM within 5 s, using faradaic currents from the redox process [85]. While not measuring blood glucose directly, this work confirmed the feasibility of the concept.

In summary, CNT-based glucose sensors combine excellent conductivity, surface area, and mechanical durability with functional versatility, enabling both enzymatic and non-enzymatic detection strategies. These platforms show high sensitivity, wide linear ranges, low LOD, and compatibility with flexible, wearable, and non-invasive applications.

#### 2.2.2. Graphene and Reduced Graphene Oxide (rGO)

Graphene and its derivatives, such as rGO, exhibit exceptional electrical conductivity, large surface area, and high electrocatalytic activity, which enhance electron transfer and sensor performance in glucose detection. GOx was immobilized on PdO NPs integrated with rGO printed on a cellulose substrate. The biosensor showed LOD of 0.046 μM, sensitivity of 0.03239 μA/μM, excellent reproducibility, stability, and selectivity, and performed effectively for glucose detection in human serum [86]. GOx immobilized on electrochemically rGO adsorbed onto poly-L-lysine-modified GCE enabled direct electron transfer and exhibited a linear range from 0.25–5 mM glucose with high electrocatalytic activity [87]. Microfabricated rGO electrodes on flexible polyimide were modified with Au-Pt alloy NPs and chitosan-GOx composites for sweat glucose detection. The sensor demonstrated sensitivity of 48 μA/mM·cm^2^, LOD of 5 μM, linear range 0–2.4 mM, and fast response (~20 s) [88]. A disposable Cu(II)/rGO-modified screen-printed carbon electrode was fabricated for cost-effective glucose detection. It exhibited high sensitivity (172 μA mM^−1^ cm^−2^), LOD of 65 μM, linear range 0.1–12.5 mM, and good anti-interference ability and reproducibility [89]. rGO nanosheets assembled on SiO_2_ nanospheres formed a 3D electroactive structure to maximize surface area for sweat glucose sensing. The NF/GOx/rGO/SiO_2_/C-PDMS biosensor achieved a sensitivity of 60.8 μA mM^−1^ cm^−2^, LOD of 3.7 μM, and a linear detection range of 0.1–9 mM [90]. A GO-modified foam nickel electrode enhanced the current response and detection sensitivity in a Pt/Ag/AgCl system. The sensor exhibited LOD of 13.3 μM, excellent linearity (R^2^ = 0.9998), and demonstrates a greener approach for blood glucose analysis [91].

Graphene-based materials, especially rGO, provide excellent platforms for both enzymatic and non-enzymatic glucose sensors. These sensors are suitable for blood and sweat glucose monitoring, including wearable applications, and can be combined with metal NPs (PdO, Au-Pt, Cu(II)) or 3D nanostructures to further boost performance.

#### 2.2.3. Carbon Quantum Dots (CQDs)

CQDs and their derivatives, such as graphene quantum dots (GQDs) and nitrogen-doped CQDs (N-CQDs), offer unique optical, electronic, and catalytic properties, making them highly suitable for both electrochemical and optical glucose sensing. GQDs were synthesized Via a low-temperature, eco-friendly hydrothermal method using citric acid. They showed high photoluminescence, water solubility, and low toxicity. Fluorescence modulation enabled glucose detection in the 2.0 × 10^−5^–2.0 × 10^−4^ M range, with a LOD of 1.53 × 10^−5^ M and binding constant of 4.05 × 10^4^. Recovery in urine samples approached 100% [92]. CNQDs were integrated with polyamide to produce a flexible wearable electrode. Pyridinic nitrogen in CNQDs enhanced conductivity and proton retention. Glucose oxidase immobilization allowed efficient glucose detection in artificial sweat, with 94.88% retention of initial performance after bending tests [93]. A fluorometric and colorimetric glucose sensor used MnO2 sheets with CQDs. Glucose oxidation generated H_2_O_2_, decomposing MnO_2_ and enhancing CQD fluorescence. Detection ranges were 5–1000 μM (fluorescent) and 5–60 μM (colorimetric), with LODs of 2.11 and 2.18 μM, respectively [94]. In situ deposition of carbon dots on CuO improved electrocatalytic activity for direct glucose oxidation. The sensor showed LOD of 1.4 nM, high sensitivity (17,142.86 μA mM^−1^ cm^−2^), and excellent long-term stability (99% current retention over 3500 s). DFT and ANN analyses confirmed glucose adsorption and detection accuracy, demonstrating potential for intelligent healthcare applications [95]. Nitrogen-doped CQDs supported on Cu nanostructures enabled sensitive fluorescence-based glucose detection. The sensor displayed a linear range of 0–140 μM and LOD of 29.85 μM, with high selectivity and sensitivity [96]. CQDs and their derivatives are versatile nanomaterials for glucose sensing, offering electrochemical and optical detection capabilities, high sensitivity, low LODs, and flexibility for wearable applications. Integration with metals (Cu, MnO_2_) or polymers enhances electrocatalytic activity, conductivity, and mechanical stability, making these sensors suitable for real-sample analysis, non-invasive sweat monitoring, and intelligent healthcare systems.

#### 2.2.4. Carbon Nanofibers (C-NFs)

C-NFs are attractive for glucose sensing due to their high surface area, porosity, and ease of functionalization, which enhance sensitivity, selectivity, and electron transport. An enzyme-free impedimetric sensor was developed using C-NFs functionalized with an aromatic diamine. EIS analysis showed high selectivity for glucose over other sugars, with a LOD of 18.64 μM [97]. Free-standing flexible C-NFs decorated with NiMoO_4_ NPs exhibited a porous architecture that accelerates glucose diffusion and improves analyte utilization. The synergistic effect of bimetallic active sites and the conductive carbon network resulted in a sensitivity of 301.77 μA mM^−1^ cm^−2^, LOD of 50 nM, and linear range of 0.3 μM–4.5 mM [98]. Glassy carbon electrodes modified with Ni(OH)_2_ nanoplates on CNFs demonstrated a wide linear range (0.001–1.2 mM), LOD of 0.76 μM, high sensitivity (1038.6 μA·mM^−1^·cm^−2^), fast response (<5 s), and excellent reproducibility [99]. CNFs combined with a novel polymer (P-BDT-BTz:BDA) on a PET substrate formed a flexible electrode for glucose detection. The sensor exhibited linear response from 20–500 μM with LOD of 8.5 μM [100]. Highly activated CNFs allow direct enzyme immobilization, producing sensitive, stable, and reproducible electrochemical biosensors [101]. Cu-Sn NPs on CNFs (CuSn/C-NFs) enhanced electrocatalytic glucose oxidation. The sensor achieved a wide linear range (0.1–9000 μM), LOD of 0.08 μM, fast response (~3 s), and good recovery in human serum [102].

CNFs provide a versatile platform for glucose sensing due to their high surface area, conductivity, and functionalization capability. Functionalization with metal oxides, bimetallic nanoparticles, or polymers, as well as enzyme immobilization, significantly improves sensitivity, selectivity, linear range, and stability, making CNF-based sensors suitable for both enzymatic and non-enzymatic electrochemical glucose detection.

### 2.3. Metal–Organic Frameworks (MOFs) Based Nanocomposites

MOF-based nanocomposites have emerged as highly advanced materials for the design and development of next-generation glucose biosensors. These materials are characterized by their highly ordered porous structures, exceptionally large surface areas, and tunable chemical and physical properties, which make them particularly suitable for applications in electrochemical glucose detection. The porous architecture of MOFs provides a vast network of channels and cavities, allowing for efficient mass transport of glucose molecules to the active sites and facilitating improved sensitivity in the sensing process. When MOFs are integrated with other functional materials such as metal NPs, carbon-based nanostructures, or enzymes, the resulting nanocomposites display significantly enhanced properties. For instance, the incorporation of conductive NPs or carbon materials improves the overall electrical conductivity of the sensor, ensuring faster electron transfer during glucose oxidation. Similarly, MOFs serve as an excellent platform for enzyme encapsulation, preserving the biological activity of immobilized enzymes while maintaining their accessibility to glucose molecules. These synergies lead to an increased number of active sites for the catalytic reaction, improved signal amplification, and reduced interference from other biomolecules, thereby enhancing the selectivity and stability of the sensor. MOF-based nanocomposites have demonstrated considerable potential in developing both enzymatic and non-enzymatic glucose sensors, offering precise, reliable, and rapid detection. The unique structural and chemical versatility of MOFs allows researchers to fine-tune the sensor performance by adjusting parameters such as pore size, functional groups, and metal centers, enabling tailored designs for specific analytical needs. Consequently, these MOF-derived nanocomposites are being widely explored for applications in personal healthcare monitoring, clinical diagnostics, and continuous glucose monitoring devices, where high sensitivity, selectivity, and long-term operational stability are critical.

#### 2.3.1. Ni-MOFs-Based Nanocomposites

Ni-MOFs, particularly three-dimensional nickel trimesic acid frameworks (3D Ni-TMAF), have attracted considerable attention for nonenzymatic glucose sensing due to their high surface area, tunable pore structures, and excellent electrochemical activity. These properties facilitate efficient electron transfer and enhance the catalytic oxidation of glucose. In one study, 3D Ni-TMAF nanospheres were constructed layer-by-layer on a highly porous nickel substrate to create an enzyme-mimicking glucose biosensor for biological solutions. The MOF, synthesized Via a solvothermal reaction of trimesic acid and nickel nitrate hexahydrate, exhibited a large surface area and abundant active sites. Electrochemical tests demonstrated a high sensitivity of 203.89 μA μM^−1^.cm^−2^, low LOD of 0.33 μM, and fast response time (<3 s), highlighting its potential as a stable and cost-effective alternative to traditional enzyme-based sensors [103]. To further enhance performance, Ag nanoparticles were incorporated into Ni-MOF nanosheets (Ag@Ni-MOF) Via a hydrothermal synthesis and chemical reduction process [104]. The 20 nm Ag NPs increased surface area, pore size, and conductivity, enhancing electrolyte diffusion. The Ag@Ni-MOF electrode achieved a specific capacitance of 1312 F/g at 1 A/g and retained 80% of its initial capacitance after 3000 charge–discharge cycles, showing higher stability than pristine Ni-MOF. As a nonenzymatic glucose sensor, it displayed a sensitivity of 160.08 μA cm^−2^.mM^−1^, LOD of 5 μM, and linear range of 5–500 μM in 0.1 M NaOH at 0.5 V. In addition to bulk MOFs, 2D Ni-MOFs provide greater exposure of active sites due to their high surface-to-volume ratio. Pyridine-regulated lamellar Ni-MOFs with ultrathin 2D morphology showed a fast amperometric response (<3 s), high sensitivity (907.54 μA mm^−1^.cm^−2^), and a wide linear range of 0.5–2665.5 μM [105]. Another high-surface-area Ni-MOF (1381 m^2^/g) with particle sizes around 80 nm achieved a linear range of 1–1600 μM, LOD of 0.76 μM, and sensitivity of 2859.95 μA mM^−1^. cm^−2^ [106].

Furthermore, ultrathin Ni-MOF nanobelts (Ni-MIL-77 NBs) were fabricated Via a one-pot solution process, offering superior glucose oxidation under alkaline conditions. Electrochemical measurements revealed a linear range of 1–500 μM, LOD of 0.25 μM, and sensitivity of 1.542 μA mM^−1^ cm^−2^ [107]. Recently, an extended gate field-effect transistor (EGFET) incorporating pyrene phosphonic acid in Ni-MOF was developed for rapid glucose detection [108]. The sensor exhibited a sensitivity of 24.5 μA mM^−1^.cm^−2^, a detection range of 20 μM–10 mM, a response time < 5 s, a LOD of 2.73 μM, and a LOQ of 8.27 μM, following Michaelis–Menten kinetics with a calculated rate constant (k_m_) of 0.07 mM, indicating a high affinity for glucose. These results demonstrate the promise of Ni-MOF-based electrodes for fast, sensitive, and reliable glucose detection in biological fluids such as saliva.

Ni-MOFs, including 3D nanospheres, Ag-doped composites, 2D lamellar structures, and ultrathin nanobelts, provide high surface area, abundant active sites, and excellent electrochemical activity, making them ideal for nonenzymatic glucose sensing (Table 5). These materials exhibit high sensitivity, low detection limits, rapid response times, and broad linear ranges. Incorporation into devices like EGFETs further enables fast, stable, and highly selective glucose detection in biological samples.

#### 2.3.2. Zn-MOF-Based Nanocomposites

A non-enzymatic glucose sensor was fabricated using electrodeposition to assemble Zn-MOF/MWCNTs on a Au electrode [109]. The sensor demonstrated improved catalytic oxidation for glucose at a low potential (0.20 V), outperforming the bare electrode (0.30 V). It exhibited a broad detection range of 0.020–8.14 mM and a LOD of 0.037 mM. The sensor also showed excellent selectivity and reliability, with recovery rates between 98.52–102.65% and a relative standard deviation of less than 8%, making it suitable for real-world applications. A MEMS system integrating electrochemical sensing and microfabrication technology was employed by Yazdan et al. to design a glucose sensor using metal–organic compounds [110]. The system incorporated a microstructure with microchannels and a three-electrode setup (reference, counter, and working electrodes). The device demonstrated high sensitivity of 35,000 μA·mM^−1^·cm^−2^ in the range 0–6 μM and a LOD of 0.18 μM, with a correlation coefficient of 0.998 (R^2^). In another study, ZnCo_2_S_4_ nanomaterials, synthesized Via static and hydrothermal methods, were used for glucose sensing. The Zn-Co bimetallic MOF precursor enhanced electrocatalytic activity, providing a LOD of 0.007 μM and two linear detection ranges: 3–9 μM and 10–100 μM for glucose [111]. A ZnCo_2_O_4_@MOF composite was developed for non-enzymatic glucose sensing [112]. The template-mediated process increased the electroactive surface area and enhanced stability. This sensor exhibited good selectivity, a wide linear range of 0.1–100 μM, and a LOD of 24.8 nM with remarkable long-term stability and reproducibility.

In summary, MOF-based glucose sensors provide ultra-sensitive, selective, and stable detection. Zn-MOF/MWCNTs offer practical and broad-range detection, MEMS-integrated systems maximize sensitivity and miniaturization, and bimetallic MOF-derived composites achieve ultralow LODs with wide linear ranges.

#### 2.3.3. Cu-MOF-Based Nanocomposites

**Figure 6:**
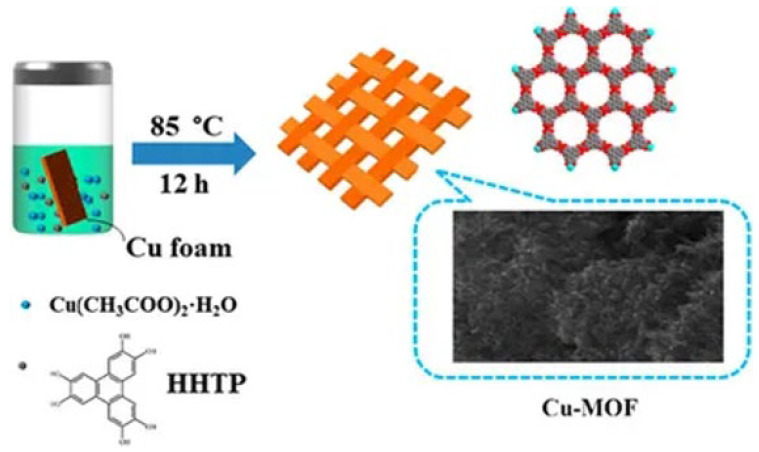
Structure and synthetic process of Cu-MOFs. Reproduced [115] under a Creative Commons Attribution 3.0 Unported Licence.

A novel porous material, Cu-hemin MOFs, was synthesized by assembling Cu^2+^ with hemin to load GO_x_ for the first time in electrochemical glucose biosensing [113]. The material exhibited excellent performance, both in oxygen reduction (Via Cu-hemin MOFs) and glucose oxidation (Via GO_x_), outperforming other GO_x_/MOFs and GO_x_/nanomaterials. As a result, the GO_x_/Cu-hemin MOFs-based glucose sensor demonstrated a wide linear range of 9.10 μM to 36.0 mM, with a LOD of 2.73 μM. In another study, a conductive Cu-MOF was synthesized from 2,3,6,7,10,11-hexahydroxytriphenylene (HHTP) ligand and copper acetate Via hydrothermal reaction [114]. The Cu-MOF demonstrated superior electrocatalytic activity for glucose oxidation under alkaline conditions. The Cu-MOF, grown on Cu foam (Cu-MOF/CF), exhibited an ultra-low LOD of 0.076 μM and a wide detection range (0.001–0.95 mM), with a high sensitivity of 30,030 mA μM^−1^ cm^−2^. Hu et al. developed a Cu-MOF from 1,2,4,5-benzene tetracarboxylic acid (H_4_BTC) using a quick, room-temperature synthesis [115]. The Cu-MOF was drop-cast onto a screen-printed carbon electrode (SPCE) for the electrochemical glucose sensor, which was further enhanced by combining it with chitosan (CS) (Figure 6). The Cs/Cu-MOF/SPCE sensor exhibited a high sensitivity of 1378.11 μA cm^−2^ mM^−1^ and an LOD of 2 μM, with a wide linear range (2 μM to 1700 μM, R^2^ = 0.992). The sensor was successfully applied to detect glucose in spiked saliva samples, with recovery percentages ranging from 95.4% to 108.7% (Table 6).

Overall, Cu-MOFs, including enzyme-loaded, conductive, and biopolymer-composite designs, offer high sensitivity, ultra-low LODs, and broad linear ranges, with proven feasibility for real-sample glucose detection.

### 2.4. Two-Dimensional Nanomaterials

#### 2.4.1. MXenes

**Figure 7:**
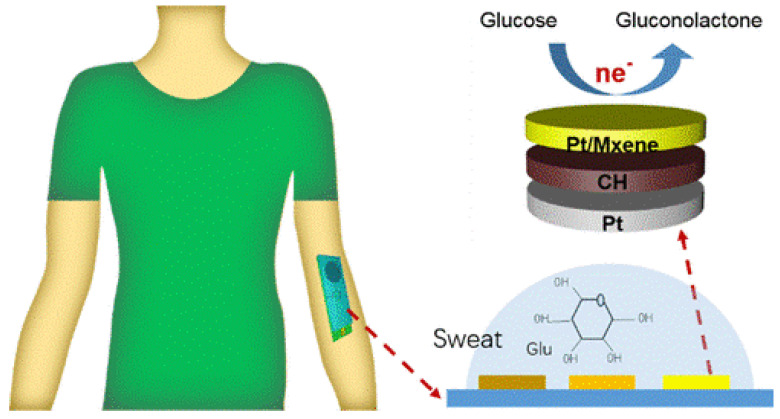
Synthesis and proposed application of Pt-loaded Ti_3_C_2_T_x_ (MXene) for glucose detection. Reprinted and Copyright American Chemical Society (2023) [117].

Titanium carbides (Ti_3_C_2_), a 2D nanomaterial family, have garnered significant attention for their unique structure and desirable properties. Their application in glucose sensing has been demonstrated in several studies (Table 7). Prabisha et al. developed a non-enzymatic glucose sensor by modifying a gold disc electrode with Nb_2_CT_x_-selenium NPs composite. The sensor exhibited an excellent glucose detection range (2–30 mM) with a sensitivity of 4.15 µA mM^−1^ cm^−2^ and a LOD of 1.1 mM at a low potential (0.16 V) in an alkaline medium [116]. A wearable, non-invasive glucose sensor was developed using Pt/MXene (Ti_3_C_2_T_x_) nanosheets, demonstrating a glucose detection range of 0–8 mmol/L under neutral conditions (Figure 7). By integrating a conductive hydrogel, the sensor achieved enhanced stability, making it suitable for continuous sweat glucose monitoring [117]. A Cu_2_O-MXene composite, synthesized Via wet precipitation, showed superior electrochemical performance compared to individual components [118]. The sensor exhibited a wide linear detection range (0.01–30 mM) and high sensitivity (11.061 µA mM^−1^ cm^−2^), with an LOD of 2.83 μM for glucose detection. Another study prepared a Cu_2_O/M/AC composite, combining MXene and CuO within a porous carbon scaffold. This composite sensor showed a dual linear detection range (0.004–13.3 mM and 15.3–28.4 mM) with an LOD of 1.96 μM and sensitivity values of 430.3 and 240.5 μA mM^−1^ cm^−2^, respectively [119]. Wensi Zhang et al. proposed a highly stretchable sensor based on a liquid metal-MXene hydrogel interface. This sensor demonstrated a low LOD of 0.77 μM, high sensitivity (1.122 μA⋅μM^−1^⋅cm^−2^), and a broad detection range (10–1000 μM) for glucose monitoring in sweat [120]. Asma Alshraim et al. developed a copper/Cuprous oxide/carbon nanoparticle-MXene composite with a wide glucose detection range (0.001–26.5 mM), a sensitivity of 762.53 μA mM^−1^ cm^−2^, and an LOD of 0.103 μM. The sensor also exhibited good stability and reusability over time [121]. A metasurface biosensor, integrating Ti_3_C_2_ MXene and graphene oxide (MG), was used for ultra-sensitive glucose detection in a non-invasive setup. The sensor achieved an LOD of 106.8 µM and significantly enhanced sensitivity through the integration of GOx and MG materials [122]. Xiaohua Zhu et al. developed a fluorescent turn-on nanosensor for glucose detection using Ti_3_C_2_ nanosheets and red-emitting carbon dots (RCDs). The sensor exhibited a linear detection range from 0.1 to 20 mM and a detection limit of 50 μM based on the fluorescence recovery due to glucose oxidation catalyzed by glucose oxidase [123].

The ZnFe Prussian blue analogue [ZnFe(PBA)] was integrated with Ti_3_C_2_Tx (MXene) Via an in situ sonication method to form ZnFe(PBA)@Ti_3_C_2_Tx, used as a non-enzymatic screen-printed electrode sensor [124]. Non-enzymatic sensors offer high sensitivity, fast response, low cost, and simple design. The cubic structure of ZnFe(PBA) provides a large surface area, while the Ti_3_C_2_ nanosheets enhance electrical conductivity, resulting in a glucose sensor with a sensitivity of 973.42 μA mM^−1^ cm^−2^, a detection limit of 3.036 μM (S/N = 3), and a linear detection range of 0.01–1 mM. Similarly, ZnFe(PBA)@Mo_3_C_2_Tx, prepared by the same method, demonstrates excellent performance as a cost-effective and biocompatible glucose sensor, achieving a sensitivity of 818.29 μA mM^−1^ cm^−2^ and a detection limit of 1.6 μM (S/N = 3) [125]. Glucose analysis in human sweat showed a strong correlation with blood glucose values, with a relative standard deviation of 2.98%.

Hence, Ti_3_C_2_ MXene-based glucose sensors combine high sensitivity, low detection limits, wide linear ranges, and flexible device integration. Non-enzymatic composites excel in high-performance, rapid-response, and cost-effective sensing, whereas enzymatic and wearable MXene platforms enable real-time, non-invasive monitoring. These features make Ti_3_C_2_ MXenes a versatile and promising material family for next-generation glucose biosensing technologies.

#### 2.4.2. MoS_2_

MoS_2_ is highly favored in glucose biosensors due to its excellent properties, enabling the creation of sensitive, selective, and stable devices for glucose detection. It serves as an ideal channel material in field-effect transistors or a catalytic support in electrochemical sensors. MoS_2_-based biosensors can be either enzymatic, using immobilized GOx, or non-enzymatic, relying on MoS_2_’s electrocatalytic properties or composites with materials like nickel oxide (NiO) or noble metals (Au, Ag, Pt) for direct glucose detection. These sensors are known for their rapid response, wide detection ranges, and potential for low-cost, real-time glucose monitoring in biological samples such as blood and urine (Table 8).

Krishna Prasad Sharma et al. prepared a CuS/MoS_2_ composite for a non-enzymatic glucose sensor, achieving a sensitivity of 252.71 μA mM^−1^ cm^−2^ and an LOD of 1.52 μM, demonstrating its effectiveness in glucose oxidation in alkaline solutions [126]. Another study explored enzyme-free glucose sensors using 2D MoS_2_ nanostructures synthesized by hydrothermal methods. The MoS_2_-GCE interface effectively detected glucose, showing a linear range of 0.01–0.20 μM with a very low LOD of 22.08 ng mL^−1^, offering a potential solution for flexible and disposable glucose sensors [127]. MoS_2_-based field-effect transistors (FETs) have gained attention for glucose detection, with sensitivity up to 260.75 mA mM^−1^ and an LOD of 300 nM. These devices demonstrate high sensitivity, rapid response times (<1 s), good stability, and a wide linear detection range (300 nM–30 mM) [128]. An organic electrochemical transistor (OECT) with MoS_2_ nanosheets modified on the gate electrode was proposed for glucose sensing. This device had a significantly improved LOD of 100 nM, 1–2 orders of magnitude better than non-modified devices [129]. P. A. Borade et al. developed a disposable carbon-based paper electrode with a NiO-MoS_2_ hybrid for glucose detection in alkaline solutions, with an LOD down to 10 μM. The catalytic enhancement of the MoS_2_-NiO hybrid was attributed to the edge sites and defects of MoS_2_, facilitating an enzyme-free oxidation reaction for glucose detection [130].

In summary, MoS_2_-based glucose biosensors combine high sensitivity, wide detection ranges, low detection limits, and stability, making them promising candidates for real-time, low-cost, and portable glucose monitoring. Among the different designs, FETs and OECTs deliver the best detection limits and rapid responses, while composite-based and nanostructured electrodes excel in cost-effectiveness and disposability. Together, these advances position MoS_2_ as a leading material in the development of next-generation glucose biosensing platforms.

#### 2.4.3. Black Phosphorus (BP)

Black phosphorus (BP), a two-dimensional (2D) material, has gained significant attention due to its unique structure and physicochemical properties, including tunable band gaps, high carrier mobility, large surface area, and chemical reactivity [131]. The large surface area of nano BP offers numerous reactive sites that enhance intralayer chemical interactions, making it a versatile scaffold for materials engineering with potential applications in chemistry, catalysis, energy, and biomedicine (Table 9).

In a biosensor design, a multilayer structure consisting of cesium fluoride (CsF), copper (Cu), halide perovskite (FASnI_3_), and 1–6 layers of BP were used to achieve exceptional performance [132]. A five-layer BP configuration enhanced electromagnetic field confinement, yielding a sensitivity over 500°/RIU and a quality factor above 140, significantly outperforming conventional surface plasmon resonance (SPR) designs. The cost-effective Cu layer and BP’s optical properties establish a superior glucose diagnostic tool.

Wearable, non-invasive glucose sensors are essential for continuous monitoring of metabolic conditions. Ecem Ezgi Özkahraman et al. developed a black phosphorus/graphitic carbon nitride (BP-gCN) heterostructure, enhancing nonenzymatic glucose oxidation activity [133]. The BP-gCN heterostructure shows an improved electrochemical surface area, nearly halving the charge transfer resistance, achieving a glucose sensitivity of 4.75 µA mM^−1^ cm^−2^ in artificial sweat. Theoretical calculations confirm stronger glucose adsorption and higher charge transfer compared to pristine gCN. This BP-gCN sensor is integrated into a wearable skin patch with a microfluidic layer and a near-field communication chip.

A new biosensor using the Kretschmann configuration for analyte detection with varying refractive indices includes a TiO_2_/SiO_2_ bi-layer sandwiched between a BK7 prism and an Ag/Au bimetallic layer, covered by BP [134]. This setup achieves a sensitivity of 240 deg/RIU and a quality factor of 34.7 RIU^−1^, with an analyte refractive index range between 1.330 and 1.347, ideal for glucose detection in urine.

Esmat Rafiee et al. proposed a biosensor based on graphene, plasmonic materials, and black phosphorus, utilizing the plasmon-induced transparency (PIT) effect for enhanced biosensing [135]. After optimizing structural parameters and chemical potential, the biosensor demonstrated excellent performance, achieving a sensitivity factor of 2686.5 nm/RIU, a figure of merit (FOM) of 134.325 RIU^−1^, and high specificity for glucose detection in blood samples, thereby aiding in the early diagnosis of diabetes.

Hence, BP-based sensors combine high sensitivity, tunable optical/electrochemical properties, and flexibility for wearable integration. Multilayer optical designs excel in refractive index detection, BP-gCN heterostructures are ideal for wearable electrochemical sensing, and PIT-based plasmonic sensors provide ultra-high sensitivity for clinical applications. Together, these approaches highlight BP as a versatile and high-performance material for next-generation glucose biosensing technologies.

### 2.5. Quantum Dots and Nanoclusters

#### 2.5.1. CdSe Quantum Dots

Chin-Ping Huang et al. developed a simple glucose detection system using GOx and CdSe/ZnS QDs, where the QDs acted as indicators to reflect changes in acidity from glucose oxidation [136]. The system exhibited a linear relationship between the quenching of photoluminescence (PL) at 586 nm and glucose concentration, in the ranges of 0.2–10 mM and 2–30 mM, depending on buffer conditions. This system enables visual glucose concentration determination by color change in QD fluorescence (Table 10). A semiconducting, water-soluble QD system (CdSe/ZnS) capped with a thiolated ligand was designed for sensitive glucose detection in aqueous samples [137]. The QDs, with a particle size of 10–12 nm and emission at 620 nm, underwent ligand-exchange modification with organic ligands. The resulting GOx:HRP/CdSe/ZnS-TGA QDs system showed strong fluorescence quenching, making it suitable for glucose detection in real samples. CdSe/ZnS core–shell QDs were conjugated with GOx and horseradish peroxidase (HRP) to create QD-FRET probes for glucose sensing [138]. The QDs acted as electron donors, while the enzymes (GOx and HRP) facilitated electron transfer, causing fluorescence quenching. The sensor demonstrated a linear detection range of 0–5.0 g/L (R^2^ = 0.992), with minimal interference from temperature, pH, and ions. A glucose sensor was developed based on Ag NPs-enhanced fluorescence of CdSe QDs [139]. The Ag NP-CdSe QD complexes exhibited up to a 9-fold fluorescence enhancement, which decreased upon interaction with glucose, correlating with glucose concentration from 2 to 52 mM, with a detection limit of 1.86 mM. Melahat et al. synthesized CdTe/ZnS core/shell QDs for detecting critical metabolites like glucose in biological samples [140]. These QDs, modified with mercaptopropionic acid, exhibited excellent quantum efficiency and stability. The sensor achieved a LOD of 0.33 mM with high linearity (R^2^ > 0.97). A new glucose sensor using fluorescence quenching of CdS QDs immobilized in glucose-sensitive microgels was developed [141]. This sensor utilized a PBA analogue for high glucose binding at physiological pH and operated within the glucose concentration range of 1–25 mM.

QD-based glucose sensors provide sensitive, stable, and versatile platforms for both enzymatic and visual detection. CdSe/ZnS systems excel in rapid, real-time fluorescence-based detection, Ag-enhanced QDs improve signal intensity for broader ranges, and CdTe/ZnS or CdS QDs offer high quantum efficiency and stability.

#### 2.5.2. Au Nanoclusters

Au nanoclusters (NCs) enhance glucose biosensors by improving electro-catalytic activity or acting as fluorescence probes that change in response to glucose (Table 11). These sensors detect hydrogen peroxide from an enzyme like GOx or use non-enzymatic glucose oxidation, with NCs facilitating electron transfer and signal generation. Their high surface area and unique optical properties enable the creation of sensitive, selective devices with low detection limits and rapid response times. One sensor design integrates Au NCs on a metallic nanotube array (MeNTA) and polypyrrole nanowire (PPyNW) [142]. Produced Via femtosecond pulse laser irradiation, the Au NCs were successfully deposited on both materials. The Au NC/MeNTA sensor demonstrated superior electrocatalytic performance for glucose oxidation, with better sensitivity, lower detection limit, broader linear range, and enhanced stability, unaffected by interfering agents like ascorbic acid and urea. To overcome enzyme immobilization challenges in electrochemical biosensors, conductive Au NCs were embedded with glucose oxidase and horseradish peroxidase to form gold nanocluster-embedded dual-enzyme NPs (Au NC-DENPs) [143]. These nanoparticles offer improved conductivity, resulting in a highly sensitive biosensor (18,944 μA/mM cm^2^) capable of detecting glucose from 5 to 320 nM, with a low detection limit of 2.58 nM. A glucose biosensor based on Au-cluster emission quenching in the UV range demonstrated high sensitivity to β-d-glucose (2.5–25.0 mM) [144]. The sensor’s practical utility was validated by measuring glucose levels in human serum, with results comparable to clinical data. Two enzymatic, mediator-free glucose biosensors were developed using premodified graphite rod (GR) electrodes [145]. The GR/DGNS/Cys/GOx and GR/DGNS/Cys/PA-AuNPs-GOx/GOx sensors showed good stability (over 71 days) and sensitivity (93.7 and 72.0 μA/mM cm^2^), with LODs of 0.027 mM and 0.034 mM, respectively.

Hence, Au NC-based glucose sensors leverage electrocatalytic and optical properties to achieve highly sensitive, selective, and stable glucose detection. Their flexibility in both electrochemical and fluorescence-based designs makes them suitable for low-concentration detection, real-sample analysis, and long-term sensor applications.

### 2.6. Hybrid Nanocomposites

#### 2.6.1. rGO–Au–ZnO or CNT–Pt–Chitosan Hybrid Nanocomposites

ZnO nanostructures and CNT-based composites have been widely explored for glucose biosensing due to their high electron transfer efficiency, biocompatibility, and catalytic activity. These materials are commonly integrated with GO_x_ or metal NPs for both enzymatic and non-enzymatic sensing.

ZnO nanorods/Au hybrid nanocomposites (ZnO/Au) were synthesized through a simple hydrothermal process, with Au nanocrystals growing on the surface of ZnO nanorods [146]. The composites exhibited excellent electron transfer properties and biocompatibility (Table 12). A glucose biosensor was fabricated by entrapping GOx within this composite matrix using glutaraldehyde and Nafion solutions. This biosensor displayed a linear response to glucose over a concentration range of 0.1–33.0 μM (R^2^ = 0.9956), with a LOD of 10 nM (S/N = 3) at an operating potential of +0.55 V in a pH 7.4 phosphate-buffered solution (PBS). It showed high sensitivity, a fast response time (within 5 s), good storage stability, and high affinity to GOx (KappM = 0.41 mM). A novel nanocomposite of binary ZnO–CoO NPs loaded on graphene nanosheets (ZnO–CoO/rGO) was successfully synthesized Via a simple two-step process [147]. These hybrids, with high electrical conductivity and abundant active sites, were modified on a GCE for glucose and H_2_O_2_ detection. The biosensor exhibited a wide linear range for glucose (10 μM to 11.205 mM) and H_2_O_2_ (25 μM to 11.1 mM), with sensitivities of 168.7 μA mM^−1^ cm^−2^ for glucose and 183.3 μA mM^−1^ cm^−2^ for H_2_O_2_. The limits of detection were 1.3 μM for glucose and 0.44 μM for H_2_O_2_. A ZnO/Co_3_O_4_/rGO nanocomposite was developed using a hydrothermal method for non-enzymatic glucose sensing [68]. This nanocomposite demonstrated excellent electrochemical performance with higher catalytic activity, lower working potential (0.55 V), and low charge transfer resistance. The electrochemical glucose sensor exhibited a wide linear range (0.015–10 mM), high sensitivity (1551.38 μA mM^−1^ cm^−2^), and low LOD (0.043 μM), along with a fast response time (~3 s). The sensor also showed good resistance to biologically interfering molecules and chloride ions, with good repeatability, reproducibility, and long-term stability. CS-core shelled CNTs were synthesized to create a hydrophilic, biocompatible composite [148]. The CS-CNT85 composite was modified with GOx and used to form a GOx-CS-CNT85/ITO electrode. The electrode showed a fast electron transfer constant (ks = 8.2 s−1). The sensor responded to increasing glucose concentrations without interference from physiological substances like ascorbic acid and uric acid. A new glucose biosensor was fabricated using multi-walled CNTs, Pt NPs, and a sol–gel CS/silica organic–inorganic hybrid composite [149]. The sensor exhibited low interference in phosphate buffer solutions (PBS, pH 6.8), a wide linear range (1.2 × 10^−6^ to 6.0 × 10^−3^ M), low LOD (3.0 × 10^−7^ M), and high sensitivity (2.08 μA mM^−1^), with a rapid response time (within 5 s) and good recovery (over 95%). An amperometric glucose biosensor, based on GOx embedded in ZnO-CS hybrid composite films on Pt-Fe(III) electrodes, was developed by Eun-Sook Paik et al. [150]. The biosensor showed a fast amperometric response (less than 10 s), a linear range of 10 μM to 11.0 mM glucose, a detection limit of 1.0 μM (S/N = 3), and a sensitivity of 30.70 μA mM^−1^.cm^−2^. The sensor also demonstrated negligible interference from substances like uric acid and ascorbic acid, and retained 87% of its initial performance after two weeks of storage at 4 °C, indicating successful immobilization of GOx with maintained enzymatic activity.

Overall, ZnO-based hybrid nanocomposites provide flexible, high-performance platforms for both enzymatic and non-enzymatic glucose sensors. Enzymatic ZnO/Au and CNT-based composites are ideal for highly selective, biocompatible sensing, while ZnO–CoO/rGO and ZnO/Co_3_O_4_/rGO hybrids offer ultra-high sensitivity, rapid response, and robust non-enzymatic operation.

#### 2.6.2. Paper-Based Sensors

A Glossy Photo-Paper (GPP)-based Screen-Printed Chemiresistive Interdigitated Electrode (SPCIDE) has been developed for enzymatic glucose sensing [151]. Using a simple, cost-effective screen-printing method, low sheet resistance interdigitated electrodes (IDEs) were printed on GPP with graphene as the conductive ink. The IDEs were then functionalized with polyaniline (PA) Via drop-casting to create a chemiresistive matrix. For selective glucose sensing, GOx enzymes and green-synthesized silver nanoparticles (GS-Ag NPs) were immobilized on the PA material. The sensor detected glucose through amperometric measurements, showing a linear response between current change and glucose concentration (198 nM to 30 mM), with a sensitivity of 291.19 µA mM^−1^.cm^−2^, and an LOD of 198 nM. The SPCIDE sensor is inexpensive, eco-friendly, bio-synthesizable, and biodegradable, offering potential for Point-of-Care (PoC) glucose testing.

A microfluidic paper-based analytical device (μPAD) was created for enzyme-free glucose quantification with colorimetric readout [152]. Au and Ag NPs acted as peroxidase-like nanozymes and colorimetric probes, respectively. Glucose oxidation by Au NPs generates H_2_O_2_, which etches the Ag NPs, inducing a color change detectable by a smartphone app. The method exhibited a linear range of 0.50–10.0 mmol L^−1^ (R^2^ = 0.9921) and an LOD of 340 μmol L^−1^. The device offers a 20-min analysis time and high selectivity, with recovery rates ranging from 98.47% to 102.34%. This enzyme-free approach provides a cost-effective alternative to traditional enzymatic glucose sensing, ideal for diabetes diagnosis.

**Figure 8:**
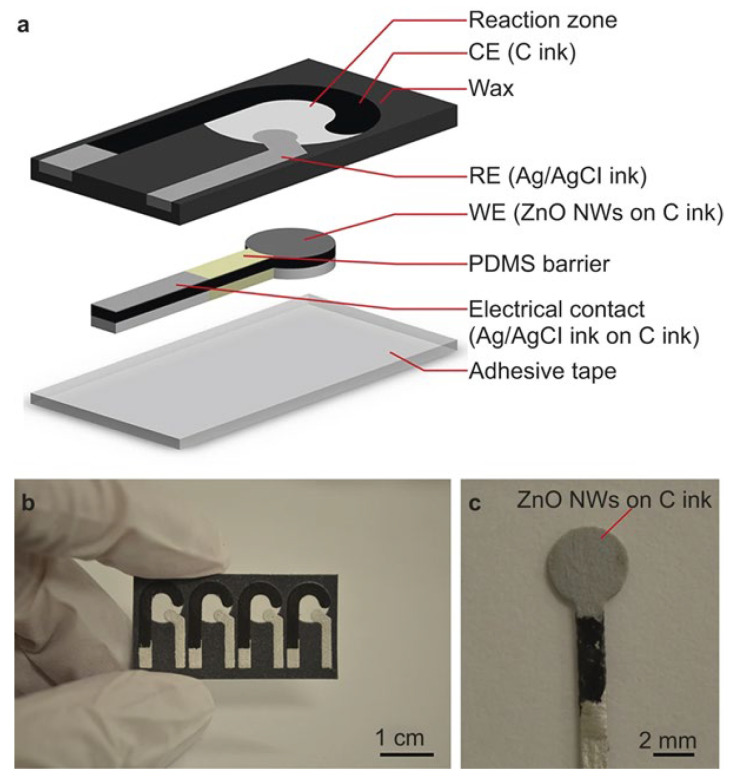
Design and fabrication of the ZnO-NW EμPAD. (**a**) Schematic of ZnO-NW EμPAD. Photographs of (**b**) an array of EμPADs and (**c**) a WE with ZnO NWs grown over its circular area (the gray color is from the ZnO NWs) [153]. Reprinted from Nature (2015) under Creative Commons Attribution 4.0 Unported License.

An electrochemical microfluidic paper-based analytical device (EμPAD) for glucose detection (Figure 8a–c), featuring a working electrode decorated with zinc oxide nanowires (ZnO NWs), was developed [153]. The EμPAD offers several advantages over traditional μPADs: (i) higher sensitivity and lower LOD due to the ZnO NWs’ high surface area and enzyme-capturing efficiency; (ii) no need for light-sensitive electron mediators, enhancing stability; and (iii) a simple, low-cost method for ZnO NW growth on paper. By adjusting the enzyme dosage and incubation time, the EμPAD achieved a sensitivity of 8.24 μA·mM^−1^·cm^−2^ with an LOD of 59.5 μM.

## 3. Emerging Trends

Biosensor technology is rapidly evolving, with several new directions shaping the future of glucose monitoring and related biomedical applications. One of the most significant developments is the integration of artificial intelligence (AI) [154]. AI-powered systems can process biosensor data in real time, enabling more accurate predictions and personalized health recommendations. For example, deep learning models applied to continuous glucose monitoring (CGM) data have been shown to predict blood sugar fluctuations, helping patients and clinicians take preventive action to avoid hypoglycemia and improve insulin management.

Another important trend is the development of smart textiles for continuous and non-invasive monitoring [155,156,157]. Fabrics embedded with conductive fibers or flexible electrodes can detect biomarkers such as glucose, lactate, and electrolytes from sweat. These textile-based sensors can be incorporated directly into clothing, making health monitoring seamless and unobtrusive. Such technologies are not only relevant for diabetes management but also for areas like sports performance tracking and occupational health.

The growing role of the Internet of Things (IoT) also offers new opportunities [158]. IoT-enabled biosensors can wirelessly transmit physiological data to cloud platforms, where advanced algorithms and AI tools provide real-time analysis and feedback. For example, smart contact lenses connected through IoT networks are being developed to measure glucose levels continuously, allowing for remote monitoring and timely medical interventions.

## 4. Summary and Conclusions

Recent research has led to the development of several innovative biosensor technologies for glucose detection, utilizing a range of nanomaterials and techniques to enhance performance, sensitivity, and usability. These include enzymatic, non-enzymatic, and hybrid approaches, often integrated into simple and cost-effective platforms. These advancements in glucose biosensing technologies highlight the potential for highly sensitive, cost-effective, and portable devices that can facilitate early diabetes detection and continuous monitoring. The integration of nanomaterials such as silver, gold, zinc oxide, and graphene significantly enhances the sensitivity, stability, and speed of glucose sensors. Together, these developments suggest that combining nanotechnology with microfluidic platforms will continue to drive innovation in glucose sensing, particularly for use in portable, affordable, and non-invasive diagnostic devices, potentially revolutionizing diabetes management and monitoring.

## 5. Future Outlook (2025+)

The future of nanosensor technology holds great promise for transforming healthcare, diagnostics, and personal wellness monitoring. One major advancement will be the use of smartphone-connected nanosensors, allowing people to continuously track multiple health parameters in real time and manage their well-being wherever they are. Combined with cloud-based diagnostics, doctors will be able to access patient data instantly, leading to faster and more accurate clinical decisions. This approach will be especially valuable in remote or underserved regions, helping to reduce inequalities in healthcare access. At the same time, the development of fully biodegradable nanobiosensors will address environmental concerns related to biomedical waste. Designed for single use, these eco-friendly devices will provide a sustainable alternative to traditional diagnostic tools while supporting responsible waste management in healthcare. Another transformative direction is the rise in AI-driven nanosensing systems [159]. Beyond simple data interpretation, AI will enable multi-parameter calibration, processing signals from different biomarkers, such as glucose, lactate, electrolytes, and stress indicators, at the same time. This will improve the accuracy and reliability of results while providing health insights that are more relevant to each individual. AI-powered calibration will also reduce sensor drift, adapt to personal variability, and improve early disease prediction, shifting care from reactive treatment to proactive prevention. The scalability of fully flexible nanosensor platforms is also an important frontier. Progress in stretchable electronics and flexible materials is paving the way for cost-effective, large-scale production of wearable and implantable sensors that naturally conform to the body. These designs will make nanosensors more comfortable, durable, and widely accessible for long-term use. To ensure these technologies can move from research to practice, the establishment of standardized evaluation protocols is essential. At present, differences in testing conditions and calibration methods make it difficult to compare results across platforms. Internationally recognized standards will provide reproducibility, regulatory approval, and clinical trust, helping nanosensors reach real-world healthcare applications more quickly.

## 6. Methodology

A comprehensive literature review was performed to gather information on glucose biosensors utilizing nanomaterials, including MOFs, 2D materials, quantum dots, carbon-based nanomaterials, and hybrid nanocomposites. Scientific articles and books were primarily retrieved from online databases such as Google Scholar, Web of Science, and Scopus, using a combination of keywords including metal-based NPs, MOFs, 2D materials, quantum dots, nanoclusters, carbon-based nanomaterials, and hybrid nanocomposites.

The selection of studies followed a systematic approach. Initially, all articles mentioning glucose biosensors were screened for relevance. Duplicates, non-English publications, and studies not focused on nanomaterial-based biosensing were excluded. The remaining studies were critically evaluated based on criteria such as type of nanomaterial, sensor design (enzymatic vs. non-enzymatic), analytical performance (sensitivity, linear range, limit of detection), device configuration, and application (e.g., blood, sweat, or urine glucose monitoring). Only studies providing sufficient experimental details or comparative performance metrics were included. References within the selected articles were also reviewed to identify additional relevant studies.

## Figures and Tables

**Table 1:** Generations of glucose biosensors.

Generations	Principle	Core Concept	Features	Limitations	Ref
First Generation (Enzymatic)	Relies on glucose oxidase (GOx) to catalyze glucose oxidation, generating hydrogen peroxide (H_2_O_2_) detected at the electrode.	GOx + O_2_ → H_2_O_2_	Simple design, directly linked to enzyme activity	Requires high operating potentials, leading to interference from electroactive species (ascorbic acid, uric acid, acetaminophen)	[10,22,23]
Second Generation (Enzymatic)	Uses redox mediators (e.g., ferrocene, quinones, metal complexes) to shuttle electrons between GOx and the electrode	Artificial mediators (e.g., ferrocene) facilitate electron transfer	Reduced applied potential, enhanced selectivity, faster response, less prone to interference	Stability issues of mediators, potential toxicity, and leaching of mediators over time	[24,25]
Third Generation (Enzymatic)	Achieves direct electrical communication between the redox center of GOx and the electrode without mediators	Direct electron transfer via nanomaterials (graphene, CNTs)– recent nanotechnology studies	High specificity, reduced background interference, real-time, and continuous monitoring	Immobilization of enzymes, maintaining enzyme activity and stability on nanostructured surfaces.	[26,27,28]
Fourth Generation (Enzymatic)	They often rely on nano-biosensing elements (nanoparticles, nanotubes, nanowires) and DNA-based sensors to enhance electron transfer and bio-recognition efficiency.	Move beyond classical enzyme–electrode systems by incorporating nanostructures and molecular probes. Enable CGM through minimally invasive or non-invasive methods.	High sensitivity and low LOD. Miniaturization for portability. Real-time, continuous monitoring with reduced sample volumes.	Biofluid variability Short sensor lifespan Interference issues Skin and wearability challenges Cost and accessibility	[29,30]
Fifth Generation (Non-enzymatic)	Built upon advances in non-invasive biosensing, wireless communication, and smart data analytics, these biosensors integrate wearable or implantable devices with digital health platforms	Fully integrated, smart biosensing systems that merge biochemical sensing with AI-driven data interpretation. Seamless wireless data transmission to smartphones, cloud platforms, or healthcare providers.	Non-invasive, painless, and continuous monitoring. Wireless communication and cloud integration for remote patient management. Personalized medicine approach. Integration with robotics and automated drug delivery systems. Predictive healthcare.	High cost and complexity Data privacy and security Dependence on infrastructure Power supply limitations User adaptability	[31,32]

**Table 2:** Different categories of sensor formats and media.

S. No	Category	Types	Important Information
1.	Sensor Formats	Electrochemical Sensors	These sensors typically use a three-electrode system (working, reference, and counter electrodes) to measure the electrical current or potential changes caused by the reaction of glucose with an enzyme or a reagent.
		Enzymatic Sensors	These sensors utilize enzymes like glucose oxidase (GOx) or glucose dehydrogenase (GDH) to catalyze the oxidation of glucose, generating a measurable signal. GOx is commonly used for detecting glucose in blood and other bodily fluids
		Amperometric Sensors	These sensors measure the current produced during the electrochemical reaction of glucose with the enzyme or reagent, providing a quantitative measurement of glucose concentration.
		Optical Sensors	These sensors use optical signals, such as fluorescence or absorbance changes, to detect glucose. Examples include sensors based on Förster Resonance Energy Transfer (FRET)
		Metal–Organic Framework (MOF) Sensors	These sensors utilize the unique properties of MOFs, such as high surface area and tunable chemistry, to enhance the sensitivity and selectivity of glucose detection.
2.	Media and Substrates	Whole Blood	Glucose biosensors are often designed to work directly with whole blood samples, offering a convenient way to monitor glucose levels in real-time.
		Interstitial fluid	CGM devices often measure glucose in the interstitial fluid, the fluid that surrounds cells, which is closely correlated with blood glucose levels
		Buffer Solutions	Enzymatic glucose sensors often utilize buffer solutions, such as phosphate buffer, to maintain a stable pH environment for the enzyme reaction.

**Table 3:** Important key points for the sensors.

Type	Materials Used	Mechanism	Selectivity	Stability	Shelf Life	Advantages	Limitations
Enzyme-Based Glucose Biosensors	Biological Component (Recognition Layer): (GOx), (GDH) Electrode/Transducer Materials: Noble metals: Pt, Au Carbon-based: Carbon nanotubes (CNTs), graphene, glassy carbon electrodes Conducting polymers: Polyaniline (PANI), Polypyrrole (PPy) Metal oxide supports: ZnO, TiO_2_, Fe_3_O_4_ nanoparticles	1. GOx mechanism (most common): Glucose + O_2_ → Gluconic acid + H_2_O_2_ The H_2_O_2_ produced is electrochemically oxidized at the electrode, generating a current proportional to glucose concentration. 2. GDH mechanism: Glucose + NAD^+^ → Gluconolactone + NADH The produced NADH can be detected electrochemically.	Very high (due to enzyme specificity)	Sensitive to pH, temperature, and humidity	Limited	High selectivity (due to enzyme specificity for glucose) High sensitivity (amplified signal due to enzyme catalysis).	Enzyme instability (sensitive to pH, temperature, and humidity). High cost due to enzyme immobilization and preservation. Limited shelf life.
Non-Enzyme-Based Glucose Biosensors	Metal Nanostructures: Platinum (Pt), Gold (Au), Palladium (Pd), Silver (Ag) Transition Metal Oxides: Copper oxide (CuO), Nickel oxide (NiO), Cobalt oxide (Co_3_O_4_), Manganese oxide (MnO_2_) Carbon-Based Materials: Graphene, CNTs, carbon nanofibers (CNFs) Hybrid Nanocomposites: Metal oxide + CNT/Graphene for synergistic catalysis	Relies on direct electrocatalytic oxidation of glucose at the electrode surface. Example with NiO: Ni^2+^ ⇌ Ni^3+^ (electrochemical activation) Glucose is oxidized to gluconolactone by Ni^3+^ Ni^3+^ is reduced back to Ni^2+^, completing the cycle. The current response corresponds to glucose concentration.	Moderate (may suffer interference)	High stability in harsh conditions	Longer	No enzyme required → improved stability and longer shelf life. Can operate in harsh conditions (temperature, pH). Cheaper and easier fabrication.	Lower selectivity (other sugars, uric acid, and ascorbic acid may interfere). Higher operating potential is often needed (may increase background signals). Sensitivity is sometimes lower than enzyme-based systems.

**Table 4:** Metal and Metal oxide nanoparticles in glucose biosensors.

Nature of NPs	NPs Used	Combinations	LOD
Metal NPs	Ag NPs	Ag NPs	
		Ag NPs capped with PVA	
		Ag NPs	0.2–100 μM
		Ag NPs decorated with ligands	0.2–100 μM
		Ag NPs with ATR optics	3.7 × 10^−4^ mol. L^−1^
	Au NPs	Au NPs with Chitosan	
		AuNPs-anchored nitrogen-doped graphene	
		Self-assembled Au NPs	
		Graphite/SrPdO3 electrode modified by Au NPs	10.1 μ.mol. L^−1^
		Modified gold electrode (Au NPs)	
		Nanoporous Au in NH_4_Cl Solution	2.5 μM
		Nanoporous Au	3 μM
		Nanoporous Au	5.0 mM
	Pt NPs	Pt NPs with Polyaniline as Hydrogel	0.7 μM
		Pl NPs/polyaniline–montmorillonite (PANI-MMT)	0.1 μM
		Hollow carbon spheres decorated with PL NPs (Pt/HCSs)	1.8 μM
		Ultrafine Pl NPs	5 μM
Metal oxide NPs	CuO NPs	CuO NPs	0.05–5 mM
		Cu_2_O/CuO NPs	0.052 μM
		CuO/MCM41	16 nm
		CuO/Graphene composite	
		CuO nanowire	0.45 μM
		CuO urchins + Cu microsperoids	
	NiO NPs	NiO NPs	0.25 μM
		NiO NPs/GCE	5.5 μM
		NiO NPs/Carbon paste electrode	0.11 μM.L^−1^
		NiO/Graphene nanosheet	5.0 μM
		NiO thin film/F-doped SnO_2_	24 μM
		Bacteria-induced hollow cylinder NiO nanomaterials	0.9 μM
		NiO NPs/functionalized SBA 15	0.023 μM
	Co_3_O_4_ NPs	Co_3_O_4_ NPs	5 μM
		Co_3_O_4_ NPs/Graphene	0.06 μM
		Co_3_O_4_ NPs/ZnO/rGO nanocomposite	0.043 μM
		Co_3_O_4_ NPs/Glassy carbon	0.15 μM
		Co_3_O_4_ NPs/Pongan seed shell activated carbon	21 nm
		Co_3_O_4_ nanoflower	0.04 μM
Bimetallic NPs	Pt-Ni	Pt-Ni modified boron-doped diamond (BDD)	
	Cu-Ni	Cu-Ni	
	Ni-Co	Ni-Co	
	Pt-Ni	Pt-Ni dispersed in Graphene	16 uM
	Cu-Ag	Cu-Ag	

**Table 5:** Ni-MOFs for glucose biosensor.

S. No	Sensor Material	Outputs
1	3D Ni-TMAF	High sensitivity (203.89 μA μM^−1^.cm^−2^), LOD 0.33 μM, <3 s response.
2	Ag@Ni-MOF	Enhanced conductivity and stability, sensitivity 160.08 μA cm^−2^.mM^−1^, LOD 5 μM.
3	2D Ni-MOFs	Ultrathin, fast response, large linear range.
4	Ni-MOF nanobelts	Efficient nonenzymatic glucose oxidation, LOD 0.25 μM.
5	Ni-MOF EGFET	Rapid, high-affinity glucose detection in saliva, following Michaelis–Menten kinetics.

**Table 6:** Cu-MOF sensor material for glucose detection.

S. No	Sensor Material	Outputs
1	Cu-hemin MOF-based sensor	Achieved a wide linear detection range (9.10 μM to 36.0 mM) and a low LOD of 2.73 μM, outperforming other GOx-based sensors.
2	Cu-MOF/CF sensor	Demonstrated an ultra-low LOD of 0.076 μM and a high sensitivity of 30,030 mA μM^−1^ cm^−2^ over a wide detection range (0.001–0.95 mM).
3	Cs/Cu-MOF/SPCE sensor	Displayed a high sensitivity of 1378.11 μA cm^−2^ mM^−1^, an LOD of 2 μM, and excellent recovery (95.4% to 108.7%) for glucose detection in saliva samples.

**Table 7:** MXene-based sensor materials for glucose detection.

S. No	Sensor Material	Outputs
1	Nb_2_CT_x_-selenium NPs sensor.	Detected glucose from 2 to 30 mM with a LOD of 1.1 mM and sensitivity of 4.15 µA mM^−1^ cm^−2^.
2	Wearable Pt/MXene sensor	Provided a glucose detection range of 0–8 mmol/L and enhanced stability Via hydrogel integration.
3	Cu_2_O-MXene composite sensor	Demonstrated a linear range (0.01–30 mM), sensitivity of 11.061 µA mM^−1^ cm^−2^, and LOD of 2.83 μM.
4	Cu_2_O/M/AC sensor	Exhibited two linear ranges (0.004–13.3 mM and 15.3–28.4 mM) with a LOD of 1.96 μM.
5	Liquid metal-MXene hydrogel sensor	Achieved a low LOD (0.77 μM) and broad glucose detection range (10–1000 μM).
6	Copper oxide-MXene composite sensor	Provided a wide glucose detection range (0.001–26.5 mM) with a sensitivity of 762.53 μA mM^−1^ cm^−2^ and LOD of 0.103 μM.
7	MGMSPR sensor (MXene-Graphene oxide)	Detected glucose with an LOD of 106.8 µM using a metasurface-based biosensor.
8	Fluorescent turn-on Ti_3_C_2_-RCDs sensor	Achieved a detection range from 0.1 to 20 mM and LOD of 50 μM.
9	ZnFe(PBA)@Ti_3_C_2_T_x_ nanohybrid	Improved sensitivity of 973.42 µA mM−1 cm−2 with the limit of detection (LOD) of 3.036 µM (S/N = 3) and linear detection range (LDR) from 0.01 to 1 mM.
10	ZnFe(PBA)@Mo_3_C_2_T_x_	Excellent analytical performance with a sensitivity of 818.285 μA mM_−1_ cm_−2_ and the limit of detection (LOD) of 1.6 μM (S/N = 3)

**Table 8:** A glucose sensor with the MoS_2_ materials.

S. No	Sensor Material	Outputs
1	CuS/MoS_2_ composite sensor	Sensitivity of 252.71 μA mM^−1^ cm^−2^, LOD of 1.52 μM for glucose oxidation in alkaline solutions.
2	MoS_2_-GCE interface sensor	Linear range of 0.01–0.20 μM and LOD of 22.08 ng mL^−1^, suitable for flexible, disposable glucose sensors.
3	MoS_2_-based FET	Sensitivity of 260.75 mA mM^−1^, LOD of 300 nM, fast response time (<1 s), wide detection range (300 nM–30 mM).
4	OECT with MoS_2_ nanosheets	LOD of 100 nM, significant improvement over non-modified devices.
5	NiO-MoS_2_ hybrid paper electrode	LOD of 10 μM, enzyme-free glucose detection through oxidation at MoS_2_-NiO hybrid sites.

**Table 9:** A glucose sensor with the BP materials.

S. No	Sensor Material	Outputs
1	BP-based Biosensor	Sensitivity > 500°/RIU, quality factor > 140 for glucose detection, leveraging the unique properties of BP in a cost-effective design.
2	BP-gCN Heterostructure	Achieved glucose sensitivity of 4.75 µA mM^−1^ cm^−2^, improved electrochemical performance, and integrated into a wearable skin patch for real-time glucose monitoring.
3	Bilayer sandwiched TiO_2_/SiO_2_ with a BK7 prism and an Ag/Au layer, covered by a BP layer	Sensitivity of 240 deg/RIU and quality factor of 34.7 RIU^−1^, suitable for glucose detection in urine with a refractive index variation of 10^−3^.
4	PIT-based Biosensor	Sensitivity factor of 2686.5 nm/RIU, FOM of 134.325 RIU^−1^, excellent performance for glucose detection, enabling early diabetes diagnosis.

**Table 10:** Quantum dot-based materials for glucose detection.

S. No	Sensor Material	Outputs
1	GOx-CdSe/ZnS QDs Sensor	Linear glucose detection ranges of 0.2–10 mM and 2–30 mM, with visual glucose concentration determination Via QD fluorescence.
2	CdSe/ZnS Core–Shell QDs Sensor	Fluorescence quenching for glucose detection, demonstrating high sensitivity and suitability for real samples.
3	GOx-HRP QD-FRET Probes	Linear detection range of 0–5.0 g/L for glucose, with minimal interference from temperature, pH, and ions.
4	Ag NPs-CdSe QDs Sensor	Fluorescence enhancement and quenching with glucose, achieving a detection limit of 1.86 mM.
5	CdTe/ZnS QDs for Metabolite Detection	High glucose sensitivity with a detection limit of 0.33 mM and excellent stability.
6	CdS QDs in Glucose-Sensitive Microgels	A fluorescence quenching glucose sensor with a range of 1–25 mM glucose.

**Table 11:** Au nanocluster-based glucose biosensors.

S. No	Sensor Material	Outputs
1	Au NCs in Biosensors	Enhance electro-catalytic activity and fluorescence detection for glucose, improving sensitivity and response times.
2	Au NCs on MeNTA and PPyNW	Demonstrates better electrocatalytic performance with a broader linear range, LOD limit, and stability against interference.
3	Au NC-DENPs	Dual-enzyme NPs offer high sensitivity (18,944 μA/mM cm^2^) and a low LOD (2.58 nM) for glucose.
4	Enzyme-Free GR Electrode Biosensors	Show good stability, sensitivity, and detection limits for glucose, with long-term performance.

**Table 12:** Various hybrid nanocomposite materials for glucose biosensors.

S. No	Sensor Material	Outputs
1	ZnO/Au Hybrid Biosensor	Exhibited high electron transfer, good biocompatibility, and a wide linear range (0.1–33.0 μM) for glucose with a low detection limit of 10 nM.
2	ZnO–CoO/rGO Nanocomposite	Showed high conductivity and active sites for multi-functional glucose and H_2_O_2_ detection, with sensitivities of 168.7 μA mM^−1^ cm^−2^ and 183.3 μA mM^−1^ cm^−2^, respectively.
3	ZnO/Co_3_O_4_/rGO Sensor	Demonstrated high sensitivity (1551.38 μA mM^−1^.cm^−2^), low LOD (0.043 μM), and fast response for glucose determination
4	CS-CNT85 Biosensor	Featured a fast electron transfer constant and stable performance without interference from physiological substances.
5	Pt NPs–CNT–CS/Silica Sensor	Achieved a wide linear range (1.2 × 10^−6^ to 6.0 × 10^−3^ M) and fast glucose detection with minimal interference.
6	GOx-ZnO/CS Composite Sensor	Showed rapid glucose detection (within 10 s) with a sensitivity of 30.70 μA mM^−1^.cm^−2^ and good storage stability.

## Data Availability

No new data were generated in this study.

## References

[1] Centers for Disease Control and Prevention (CDC) (2024). National Diabetes Statistics Report. https://www.cdc.gov/diabetes/php/data-research/methods.html?CDC_AAref_Val=https://www.cdc.gov/diabetes/data/statistics-report/index.html.

[2] International Diabetes Federation: Brussels, 2025. https://diabetesatlas.org/#:~:text=The%20International%20Diabetes%20Federation%20%28IDF%29%20is%20an%20umbrella,national%20diabetes%20associations%20in%20158%20countries%20and%20territories.

[3] Mittal R., Koutras N., Maya J., Lemos J.R.N., Hirani K. (2024). Blood glucose monitoring devices for type 1 diabetes: A journey from the food and drug administration approval to market availability. Front. Endocrinol..

[4] Ogle G.D., Wang F., Haynes A., Gregory G.A., King T.W., Deng K., Dabelea D., James S., Jenkins A.J., Li X. (2025). Global type 1 diabetes prevalence, incidence, and mortality estimates 2025: Results from the International Diabetes Federation Atlas, 11th Edition, and the T1D Index Version 3.0. Diabetes Res. Clin. Pract..

[5] Libman I., Haynes A., Lyons S., Pradeep P., Rwagasor E., Tung J.Y.-L., Jefferies C.A., Oram R.A., Dabelea D., Craig M.E. (2022). ISPAD Clinical Practice Consensus Guidelines 2022: Definition, epidemiology, and classification of diabetes in children and adolescents. Pediatr. Diabetes.

[6] Tomic D., Harding J.L., Jenkins A.J., Shaw J.E., Magliano D.J. (2025). The epidemiology of type 1 diabetes mellitus in older adults. Nat. Rev. Endocrinol..

[7] Reed J., Bain S., Kanamarlapudi V. (2021). A Review of Current Trends with Type 2 Diabetes Epidemiology, Aetiology, Pathogenesis, Treatments and Future Perspectives. Diabetes Metab. Syndr. Obes. Targets Ther..

[8] Harding J.L., Wander P.L., Zhang X., Li X., Karuranga S., Chen H., Sun H., Xie Y., Oram R.A., Magliano D.J. (2022). The incidence of adult-onset type 1 diabetes: A systematic review from 32 countries and regions. Diabetes Care.

[9] Iqbal S., Jayyab A.A., Alrashdi A.M., Reverté-Villarroya S. (2023). The predictive ability of C-peptide in distinguishing type 1 diabetes from type 2 diabetes: A systematic review and meta-analysis. Endocr. Pract..

[10] Yoo E.-H., Lee S.-Y. (2010). Glucose Biosensors: An Overview of Use in Clinical Practice. Sensors.

[11] Pullano S.A., Greco M., Bianco M.G., Foti D., Brunetti A., Fiorillo A.S. (2022). Glucose biosensors in clinical practice: Principles, limits and perspectives of currently used devices. Theranostics.

[12] Bender C., Vestergaard P., Cichosz S.L. (2025). The History, Evolution and Future of Continuous Glucose Monitoring (CGM). Diabetology.

[13] Oriot P., Klipper dit Kurz N., Ponchon M., Weber E., Colin I.M., Philips J.C. (2023). Benefits and limitations of hypo/hyperglycemic alarms associated with continuous glucose monitoring in individuals with diabetes. Diabetes Epidemiol. Manag..

[14] Cappon G., Vettoretti M., Sparacino G., Facchinetti A. (2019). Continuous glucose monitoring sensors for diabetes management: A review of technologies and applications. Diabetes Metab. J..

[15] Tummalapalli M., Singh S., Sanwaria S., Gurave P.M. (2022). Design and development of advanced glucose biosensors via tuned interactions between marine polysaccharides and diagnostic elements—A survey. Sens. Int..

[16] Johnston L., Wang G., Hu K., Qian C., Liu G. (2021). Advances in biosensors for continuous glucose monitoring towards wearables. Front. Bioeng. Biotechnol..

[17] Harun-Or-Rashid M., Aktar M.N., Preda V., Nasiri N. (2024). Advances in electrochemical sensors for real-time glucose monitoring. Sens. Diagn..

[18] Ambaye A.D., Mamo M.D., Zigyalew Y., Mengistu W.M., Fito Nure J., Mokrani T., Ntsendwana B. (2024). The development of carbon nanostructured biosensors for glucose detection to enhance healthcare services: A review. Front. Sens..

[19] Clark L.C. (1959). Electrochemical Device for Chemical Analysis. U.S. Patent.

[20] Juska V.B., Pemble M.E. (2020). A Critical Review of Electrochemical Glucose Sensing: Evolution of Biosensor Platforms Based on Advanced Nanosystems. Sensors.

[21] Malik S., Singh J., Goyat R., Saharan Y., Chaudhry V., Umar A., Ibrahim A.A., Akbar S., Ameen S., Baskoutas S. (2023). Nanomaterials-based biosensor and their applications: A review. Heliyon.

[22] Clark L.J., Lyons C. (1962). Electrode systems for continuous monitoring in cardiovascular surgery. Ann. N. Y. Acad. Sci..

[23] Lee T.M.-H. (2008). Over-the-Counter Biosensors: Past, Present, and Future. Sensors.

[24] Mohamad Nor N., Ridhuan N.S., Abdul Razak K. (2022). Progress of Enzymatic and Non-Enzymatic Electrochemical Glucose Biosensor Based on Nanomaterial-Modified Electrode. Biosensors.

[25] Huang Q., Chen J., Zhao Y., Huang J., Liu H. (2025). Advancements in electrochemical glucose sensors. Talanta.

[26] Yuwen T., Shu D., Zou H., Yang X., Wang S., Zhang S., Liu Q., Wang X., Wang G., Zhang Y. (2023). Carbon nanotubes: A powerful bridge for conductivity and flexibility in electrochemical glucose sensors. J. Nanobiotechnology.

[27] Liu Y., Zhang J., Cheng Y., Jiang S.P. (2018). Effect of Carbon Nanotubes on Direct Electron Transfer and Electrocatalytic Activity of Immobilized Glucose Oxidase. ACS Omega.

[28] Zafar H., Channa A., Jeoti V., Stojanović G.M. (2022). Comprehensive Review on Wearable Sweat-Glucose Sensors for Continuous Glucose Monitoring. Sensors.

[29] Naikoo G.A., Arshad F., Hassan I.U., Omar F.B., Tambuwala M.M., Mustaqeem M., Saleh T.A. (2023). Trends in bimetallic nanomaterials and methods for fourth-generation glucose sensors. TrAC Trends Anal. Chem..

[30] Naikoo G.A., Awan T., Salim H., Arshad F., Hassan I.U., Pedram M.Z., Ahmed W., Faruck H.L., Aljabali A.A.A., Mishra V. (2021). Fourth-generation glucose sensors composed of copper nanostructures for diabetes management: A critical review. Bioeng. Transl. Med..

[31] Wang M., Zheng J., Zhang G., Lu S., Zhou J. (2025). Wearable Electrochemical Glucose Sensors for Fluid Monitoring: Advances and Challenges in Non-Invasive and Minimally Invasive Technologies. Biosensors.

[32] Jamshidnejad-Tosaramandani T., Kashanian S., Omidfar K., Schiöth H.B. (2025). The Role of Nanomaterials in the Wearable Electrochemical Glucose Biosensors for Diabetes Management. Biosensors.

[33] Si P., Huang Y., Wang T., Ma J. (2013). Nanomaterials for electrochemical non-enzymatic glucose biosensors. RSC Adv..

[34] Tian K., Prestgard M., Tiwari A. (2014). A review of recent advances in nonenzymatic glucose sensors. Mater. Sci. Eng. C.

[35] Rahis U. (2018). Nanomaterials and Their Applications. Metal Nanoparticles as Glucose.

[36] Ren X., Meng X., Chen D., Tang F., Jiao J. (2005). Using silver nanoparticle to enhance current response of biosensor. Biosens. Bioelectron..

[37] Serra A., Filippo E., Re M., Palmisano M., Vittori-Antisari M., Buccolieri A., Manno D. (2009). Non-functionalized silver nanoparticles for a localized surface plasmon resonance-based glucose sensor. Nanotechnology.

[38] Xia Y., Ye J., Tan K., Wang J., Yang G. (2013). Colorimetric visualization of glucose at the submicromole level in serum by a homogenous silver nanoprism-glucose oxidase system. Anal. Chem..

[39] Ghiaci M., Tghizadeh M., Ensafi A.A., Zandi-Atashbar N., Rezaei B. (2016). Silver nanoparticles decorated anchored type ligands as new electrochemical sensors for glucose detection. J. Taiwan Inst. Chem. Eng..

[40] Li D., Yu S., Sun C., Zou C., Yu H., Xu K. (2015). U-shaped fiber-optic ATR sensor enhanced by silver nanoparticles for continuous glucose monitoring. Biosens. Bioelectron..

[41] Feng D., Wang F., Chen Z. (2009). Electrochemical glucose sensor based on one-step construction of gold nanoparticle-chitosan composite film. Sens. Actuators B Chem..

[42] Thanh T.D., Balamurugan J., Lee S.H., Kim N.H., Lee J.H. (2016). Effective seed-assisted synthesis of gold nanoparticles anchored nitrogen-doped graphene for electrochemical detection of glucose and dopamine. Biosens. Bioelectron..

[43] Jena B.K., Raj C.R. (2006). Enzyme-free amperometric sensing of glucose by using gold nanoparticles. Chem. A Eur. J..

[44] El-Ads E.H., Galal A., Atta N.F. (2015). Electrochemistry of glucose at gold nanoparticles modified graphite/SrPdO3 electrode—towards a novel non-enzymatic glucose sensor. J. Electroanal. Chem..

[45] Zhang S., Wang N., Yu H., Niu Y., Sun C. (2005). Covalent attachment of glucose oxidase to an Au electrode modified with gold nanoparticles for use as glucose biosensor. Bioelectrochemistry.

[46] Zhou C., Xia Y., Huang W., Li Z. (2014). A Rapid Anodic Fabrication of Nanoporous Gold in NH4Cl Solution for Nonenzymatic Glucose Detection. J. Electrochem. Soc..

[47] Chen L.Y., Lang X.Y., Fujita T., Chen M.W. (2011). Nanoporous gold for enzyme-free electrochemical glucose sensors. Scr. Mater..

[48] Mie Y., Katagai S., Ikegami M. (2020). Electrochemical Oxidation of Monosaccharides at Nanoporous Gold with Controlled Atomic Surface Orientation and Non-Enzymatic Galactose Sensing. Sensors.

[49] Zhai D., Liu B., Shi Y., Pan L., Wang Y., Li W., Zhang R., Yu G. (2013). Highly Sensitive Glucose Sensor Based on Pt Nanoparticle/Polyaniline Hydrogel Heterostructures. ACS Nano.

[50] Zheng H., Liu M., Yan Z., Chen J. (2020). Highly selective and stable glucose biosensor based on incorporation of platinum nanoparticles into polyaniline-montmorillonite hybrid composites. Microchem. J..

[51] Luhana C., Bo X.-J., Ju J., Guo L.-P. (2012). A novel enzymatic glucose sensor based on Pt nanoparticles-decorated hollow carbon spheres-modified glassy carbon electrode. J. Nanoparticle Res..

[52] Cao Z., Zou Y., Xiang C., Sun L.-X., Xu F. (2007). Amperometric Glucose Biosensor Based on Ultrafine Platinum Nanoparticles. Anal. Lett..

[53] Fan B., Spindler B.D., Zhao W., Chan H., Wang Z., Kim M., Chipangura Y., Bühlmann P., Stein A. (2023). Comparison of Copper(II) Oxide Nanostructures with Different Morphologies for Nonenzymatic Glucose Sensing. ACS Appl. Nano Mater..

[54] Amirsoleimani A.R., Siampour H., Abbasian S., Rad G.B., Moshaii A., Zaradshan Z. (2023). Copper oxide nanocolumns for high-sensitive non-enzymatic glucose sensing. Sens. Bio-Sens. Res..

[55] Arif D., Hassan M., Abdullah M., Miran W., Nasir M.A., Batool S., Baig M.A., Liaqat U. (2024). An electrochemical sensor based on copper oxide nanoparticles loaded on a mesoporous MCM-41 for non-enzymatic detection of glucose. Ceram. Int..

[56] Li Q., Yang D., Gao Y., Wang Z., Yin R., Xuan F. (2023). Highly Sensitive and Flexible Copper Oxide/Graphene Non-Enzymatic Glucose Sensor by Laser Direct Writing. Adv. Sens. Res..

[57] Jasim H.A., Dakhil O.A.A. (2022). Highly sensitive non-enzymatic glucose sensor based on copper oxide nanorods. J. Nanoparticle Res..

[58] Mamleyev E.R., Weidler P.G., Nefedov A., Szabó D.V., Islam M., Mager D., Korvink J.G. (2021). Nano-and Microstructured Copper/Copper Oxide Composites on Laser-Induced Carbon for Enzyme-Free Glucose Sensors. ACS Appl. Nano Mater..

[59] Ahamad N., Banerjee S., Wei C.-C., Lu K.-C., Khedulkar A.P., Jian W.-B., Mahmood S., Chu C.-W., Lin H.-C. (2024). Flexible Non-Enzymatic Glucose Sensors: One-Step Green Synthesis of NiO Nanoporous Films via an Electro-Exploding Wire Technique. ACS Appl. Mater. Interfaces.

[60] Venkadesh A., Mathiyarasu J., Dave S., Radhakrishnan S. (2021). Amine mediated synthesis of nickel oxide nanoparticles and their superior electrochemical sensing performance for glucose detection. Inorg. Chem. Commun..

[61] Erdem C., Koyuncu Zeybek D., Aydoğdu G., Zeybek B., Pekyardımcı Ş., Kılıç E. (2014). Electrochemical glucose biosensor based on nickel oxide nanoparticle-modified carbon paste electrode. Artif. Cells Nanomed. Biotechnol..

[62] Zeng G., Li W., Ci S., Jia J., Wen Z. (2016). Highly Dispersed NiO Nanoparticles Decorating Graphene Nanosheets for Non-enzymatic Glucose Sensor and Biofuel Cell. Sci. Rep..

[63] Naderi Asrami P., Tehrani M.S., Azar P.A., Mozaffari S.A. (2017). Impedimetric glucose biosensor based on nanostructure nickel oxide transducer fabricated by reactive RF magnetron sputtering system. J. Electroanal. Chem..

[64] Vaidyanathan S., Cherng J.-Y., Sun A.-C., Chen C.-Y. (2016). Bacteria-Templated NiO Nanoparticles/Microstructure for an Enzymeless Glucose Sensor. Int. J. Mol. Sci..

[65] Baghayeri M., Sedrpoushan A., Mohammadi A., Heidari M. (2017). A non-enzymatic glucose sensor based on NiO nanoparticles/functionalized SBA 15/MWCNT-modified carbon paste electrode. Ionics.

[66] Kalkozova Z.K., Balgimbayeva U.A., Gabdullin M.T., Gritsenko L.V., Suo G., Abdullin K.A. (2025). A Facile Method for Synthesizing Cobalt Oxide Nanoparticles to Create a Highly Sensitive Non-Enzyme Glucose Sensor. Biosensors.

[67] Karuppiah C., Palanisamy S., Chen S.-M., Veeramani V., Periakaruppan P. (2014). A novel enzymatic glucose biosensor and sensitive non-enzymatic hydrogen peroxide sensor based on graphene and cobalt oxide nanoparticles composite modified glassy carbon electrode. Sens. Actuators B Chem..

[68] Hussein B.A., Tsegaye A.A., Shifera G., Taddesse A.M. (2023). A sensitive non-enzymatic electrochemical glucose sensor based on a ZnO/Co3O4/reduced graphene oxide nanocomposite. Sens. Diagn..

[69] Sattarahmady N., Heli H. (2012). A non-enzymatic amperometric sensor for glucose based on cobalt oxide nanoparticles. J. Exp. Nanosci..

[70] Madhu R., Veeramani V., Chen S.-M., Manikandan A., Lo A.-Y., Chueh Y.-L. (2015). Honeycomb-like porous carbon–cobalt oxide nanocomposite for high-performance enzymeless glucose sensor and supercapacitor applications. ACS Appl. Mater. Interfaces.

[71] Mondal S., Madhuri R., Sharma P.K. (2017). Probing the shape-specific electrochemical properties of cobalt oxide nanostructures for their application as selective and sensitive non-enzymatic glucose sensors. J. Mater. Chem. C.

[72] Zhu R., Zhao Z., Cao J., Li H., Ma L., Zhou K., Yu Z., Wei Q. (2022). Effect of Pt-Ni deposition sequence on the bimetal-modified boron-doped diamond on catalytic performance for glucose oxidation in neutral media. J. Electroanal. Chem..

[73] Ghosh R., Li X., Yates M.Z. (2024). Nonenzymatic glucose sensor using bimetallic catalysts. ACS Appl. Mater. Interfaces.

[74] Liu M., Gao T., Li H., Xie B., Hu C., Guo Y., Xiao D. (2023). Preparation of amorphous Ni/Co bimetallic nanoparticles to enhance the electrochemical sensing of glucose. Microchem. J..

[75] Li R., Deng X., Xia L. (2020). Non-enzymatic sensor for determination of glucose based on PtNi nanoparticles decorated graphene. Sci. Rep..

[76] Rahmanipour M., Siampour H., Amirsoleimani A.R., Rezazadeh M., Moshaii A. (2023). Cu–Ag bimetallic nanostructures with high glucose sensing performance fabricated by a scalable and reproducible method. Microchem. J..

[77] Gupta P., Gupta V.K., Huseinov A., Rahm C.E., Gazica K., Alvarez N.T. (2021). Highly sensitive non-enzymatic glucose sensor based on carbon nanotube microelectrode set. Sens. Actuators B: Chem..

[78] Muqaddas S., Javed M., Nadeem S., Asghar M.A., Haider A., Ahmad M., Ashraf A.R., Nazir A., Iqbal M., Alwadai N. (2023). Carbon nanotube fiber-based flexible microelectrode for electrochemical glucose sensors. ACS Omega.

[79] Bolaños-Mendez D., Fernández L., Uribe R., Cunalata-Castro A., González G., Rojas I., Chico-Proano A., Debut A., Celi L.A., Espinoza-Montero P. (2024). Evaluation of a non-enzymatic electrochemical sensor based on Co(OH)2-functionalized carbon nanotubes for glucose detection. Sensors.

[80] Pourasl A.H., Ahmadi M.T., Rahmani M., Chin H.C., Lim C.S., Ismail R., Tan M.L.P. (2014). Analytical modeling of glucose biosensors based on carbon nanotubes. Nanoscale Res. Lett..

[81] Lin Y., Lu F., Tu Y., Ren Z. (2004). Glucose biosensors based on carbon nanotube nanoelectrode ensembles. Nano Lett..

[82] Lee H., Hong Y.J., Baik S., Hyeon T., Kim D.-H. (2018). Enzyme-based glucose sensor: From invasive to wearable device. Adv. Healthc. Mater..

[83] Rdest M., Janas D. (2021). Carbon nanotube wearable sensors for health diagnostics. Sensors.

[84] Chen Y.-S., Huang J.-H., Chuang C.-C. (2009). Glucose biosensor based on multiwalled carbon nanotubes grown directly on Si. Carbon.

[85] Kang B.-C., Park B.-S., Ha T.-J. (2019). Highly sensitive wearable glucose sensor systems based on functionalized single-wall carbon nanotubes with glucose oxidase-Nafion composites. Appl. Surf. Sci..

[86] Chen L., Zhang Y., Hu T. (2024). A graphene oxide-modified biosensor for non-invasive glucose monitoring in college athletes. Alex. Eng. J..

[87] Hua L., Wu X., Wang R. (2012). Glucose sensor based on an electrochemical reduced graphene oxide–poly(l-lysine) composite film modified GC electrode. Analyst.

[88] Xuan X., Yoon H.S., Park J.Y. (2018). A wearable electrochemical glucose sensor based on simple and low-cost fabrication supported micro-patterned reduced graphene oxide nanocomposite electrode on flexible substrate. Biosens. Bioelectron..

[89] Phetsang S., Kidkhunthod P., Chanlek N., Jakmunee J., Mungkornasawakul P., Ounnunkad K. (2021). Copper/reduced graphene oxide film modified electrode for non-enzymatic glucose sensing application. Sci. Rep..

[90] Xu M., Zhu Y., Gao S., Zhang Z., Gu Y., Liu X. (2021). Reduced graphene oxide-coated silica nanospheres as flexible enzymatic biosensors for detection of glucose in sweat. ACS Appl. Nano Mater..

[91] Chang L., Liu Y., Tian R., Meng L., Zhang H., Gao Y., Zhang F., Ruan X., Zhu B., Li J. (2020). Rapid glucose detection using graphene oxide modified foam nickel electrode with optimized basic solution. Int. J. Food Prop..

[92] Rajmane S.P., Nille O.S., Kolekar G.B., Sadale S.B. (2025). Studies on glucose detection using graphene quantum dots prepared by hydrothermal method. Sci. Rep..

[93] Chiu Y.-H., Rinawati M., Chang L.-Y., Guo Y.-T., Chen K.-J., Chiu H.-C., Lin Z.-H., Huang W.-H., Haw S.-C., Yeh M.-H. (2025). Carbon nitride quantum dots/polyaniline nanocomposites for non-invasive glucose monitoring using wearable sweat biosensor. ACS Appl. Nano Mater..

[94] Wang C., Tan R., Li L., Liu D. (2019). Dual-modal colorimetric and fluorometric method for glucose detection using MnO_2_ sheets and carbon quantum dots. Chem. Res. Chin. Univ..

[95] Khan N.U., Sharma B.P., Tumrani S.H., Zahoor M., Soomro R.A., Küçükdeniz T., Karakuş S., Elsharkawy E.R., Lu J., El-Bahy S.M. (2024). Enhanced detection of glucose with carbon quantum dot-modified copper oxide: Computational insight and machine learning modeling of electrochemical sensing. Microchem. J..

[96] Aygun A., Ozveren E., Halvaci E., Ikballi D., Elhouda Tiri R.N., Catal C., Bekmezci M., Ozengul A., Kaynak I., Sen F. (2024). The performance of a very sensitive glucose sensor developed with copper nanostructure-supported nitrogen-doped carbon quantum dots. RSC Adv..

[97] Ferlazzo A., Celesti C., Iannazzo D., Ampelli C., Giusi D., Costantino V., Neri G. (2024). Functionalization of carbon nanofibers with an aromatic diamine: Toward a simple electrochemical-based sensing platform for the selective sensing of glucose. ACS Omega.

[98] Rani S.D., Ramachandran R., Sheet S., Aziz M.A., Lee Y.S., Al-Sehemi A.G., Pannipara M., Xia Y., Tsai S.-Y., Ng F.-L. (2020). NiMoO_4_ nanoparticles decorated carbon nanofiber membranes for the flexible and high performance glucose sensors. Sens. Actuators B Chem..

[99] Zhang L., Yuan S., Lu X. (2014). Amperometric nonenzymatic glucose sensor based on a glassy carbon electrode modified with a nanocomposite made from nickel(II) hydroxide nanoplates and carbon nanofibers. Microchim. Acta.

[100] Bulut U., Sayin V.O., Altin Y., Cevher S.C., Cirpan A., Bedeloglu A.C., Soylemez S. (2023). A flexible carbon nanofiber and conjugated polymer-based electrode for glucose sensing. Microchim. Acta.

[101] Vamvakaki V., Tsagaraki K., Chaniotakis N. (2006). Carbon nanofiber-based glucose biosensor. Anal. Chem..

[102] Huan K., Li Y., Deng D., Wang H., Wang D., Li M., Luo L. (2022). Composite-controlled electrospinning of CuSn bimetallic nanoparticles/carbon nanofibers for electrochemical glucose sensor. Appl. Surf. Sci..

[103] Pavadai R., Arivazhagan M., Jakmunee J., Pavadai N., Palanisamy R., Honnu G., Kityakarn S., Khumphon J., Issro C., Khamboonrueang D. (2025). Highly porous 3D Ni-MOFs as an efficient and enzyme-mimic electrochemical sensing platform for glucose in real samples of sweat and saliva in biomedical applications. ACS Omega.

[104] Cao J., Yun J., Zhang N., Wei Y., Yang H., Xu Z. (2021). Bifunctional Ag@Ni-MOF for high performance supercapacitor and glucose sensor. Synth. Met..

[105] Zhang Q., Li P., Wu J., Peng Y., Pang H. (2023). Pyridine-regulated lamellar nickel-based metal–organic framework (Ni-MOF) for nonenzymatic electrochemical glucose sensor. Adv. Sci..

[106] Zeraati M., Alizadeh V., Kazemzadeh P., Safinejad M., Kazemian H., Sargazi G. (2022). A new nickel metal organic framework (Ni-MOF) porous nanostructure as a potential novel electrochemical sensor for detecting glucose. J. Porous Mater..

[107] Xiao X., Zheng S., Li X., Zhang G., Guo X., Xue H., Pang H. (2017). Facile synthesis of ultrathin Ni-MOF nanobelts for high-efficiency determination of glucose in human serum. J. Mater. Chem. B.

[108] Matada M.S.S., Kuppuswamy G.P., Sasi S., Jayaraman S.V., Nutalapati V., Kumar S.S., Sivalingam Y. (2024). Pyrene derivative incorporated Ni-MOF as an enzyme mimic for noninvasive salivary glucose detection toward diagnosis of diabetes mellitus. ACS Appl. Mater. Interfaces.

[109] Xue Y.-T., Chen Z., Chen X., Han G.-C., Feng X.-Z., Kraatz H.-B. (2024). Enzyme-Free Glucose Sensor Based on Electrodeposition of Multi-Walled Carbon Nanotubes and Zn-Based Metal Framework-Modified Gold Electrode at Low Potential. Electrochim. Acta.

[110] Firouzi Jahantigh Y., Mehrpooya M., Askari Moghadam R., Ganjali M.R. (2025). Design and Fabrication of Glucose Sensor Using Metal-Organic Framework Nanomaterials. Mater. Chem. Phys..

[111] Wang Y., Yan J., Zhu W., Zhang Y., Cao K., Zhang B., Yu X., Shen Q., Liu C., Wang Q. (2023). Metal-Organic Framework Derived Porous Hollow ZnCo_2_S_4_ Improving Electrocatalytic Oxidation of Glucose. Mater. Res. Express.

[112] Divyarania K., Sreenivasa S., Kumar S., Vinod A., Alharethy F., Jeon B.-H., Devie V.S.A., Martis P., Parashuram L. (2024). Fabrication of a Novel MOF Template-Derived ZnCo_2_O_4_ Composite for the Non-Enzymatic Electrochemical Detection of Glucose. Results Chem..

[113] He J., Yang H., Zhang Y., Yu J., Miao L., Song Y., Wang L. (2016). Smart Nanocomposites of Cu-Hemin Metal-Organic Frameworks for Electrochemical Glucose Biosensing. Sci. Rep..

[114] Guesmi S., Moulaee K., Bressi V., Kahri H., Khaskhoussi A., Espro C., Barhoumi H., Neri G. (2024). Non-Enzymatic Amperometric Glucose Sensing by Novel Cu-MOF Synthesized at Room Temperature. Mater. Adv..

[115] Hu Q., Qin J., Wang X.-F., Ran G.-Y., Wang Q., Liu G.-X., Ma J.-P., Ge J.-Y., Wang H.-Y. (2021). Cu-Based Conductive MOF Grown In Situ on Cu Foam as a Highly Selective and Stable Non-Enzymatic Glucose Sensor. Front. Chem..

[116] Prabisha K.E., Neena P.K., Ankitha M., Rasheed P.A., Suneesh P.V., Babu T.G.S. (2025). Selenium Nanoparticles Modified Niobium MXene for Non-Enzymatic Detection of Glucose. Sci. Rep..

[117] Li Q.-F., Chen X., Wang H., Liu M., Peng H.-L. (2023). Pt/MXene-Based Flexible Wearable Non-Enzymatic Electrochemical Sensor for Continuous Glucose Detection in Sweat. ACS Appl. Mater. Interfaces.

[118] Gopal T.S., Jeong S.K., Alrebdi T.A., Pandiaraj S., Alodhayb A., Muthuramamoorthy M., Grace A.N. (2022). MXene-Based Composite Electrodes for Efficient Electrochemical Sensing of Glucose by Non-Enzymatic Method. Mater. Today Chem..

[119] Gopal T.S., James J.T., Gunaseelan B., Ramesh K., Raghavan V., Malathi C.J., Amarnath A.K., Kumar V.G., Rajasekaran S.J., Pandiaraj S. (2024). MXene-Embedded Porous Carbon-Based Cu_2_O Nanocomposites for Non-Enzymatic Glucose Sensors. ACS Omega.

[120] Zhang W., Jiang S., Yu H., Feng S., Zhang K. (2025). Ga@MXene-Based Flexible Wearable Biosensor for Glucose Monitoring in Sweat. iScience.

[121] Alshraim A., Gopal T.S., Alanazi N., Muthumareeswaran M., Alobaidi A.A.E., Alsaigh R., Aldosary M., Pandiaraj S., Grace A.N., Alodhayb A.N. (2024). Cu/Cu_2_O/C Nanoparticles and MXene Based Composite for Non-Enzymatic Glucose Sensors. Nanotechnology.

[122] Li R., Fan H., Chen Y., Yin S., Liu G.L., Li Y., Huang L. (2025). MXene–Graphene Oxide Heterostructured Films for Enhanced Metasurface Plasmonic Biosensing in Continuous Glucose Monitoring. Adv. Sci..

[123] Zhu X., Pang X., Zhang Y., Yao S. (2019). Titanium Carbide MXenes Combined with Red-Emitting Carbon Dots as a Unique Turn-On Fluorescent Nanosensor for Label-Free Determination of Glucose. J. Mater. Chem. B.

[124] Ravitchandiran A., AlGarni S., AlSalhi M.S., Rajaram R., Malik T., Angaiah S. (2024). ZnFe(PBA)@Ti3C2Tx nanohybrid-based highly sensitive non-enzymatic electrochemical sensor for the detection of glucose in human sweat. Sci. Rep..

[125] Ravitchandiran A., Dhandapani P., Rajendra S.P., AlSalhi M.S., Rajamohan R., Angaiah S. (2024). Development of ZnFe(PBA)@Mo3C2Tx Nanohybrid based Non-Enzymatic Sensor for the Detection of Glucose in Human Sweat. Chemistry Select.

[126] Sharma K.P., Shin M., Awasthi G.P., Cho S., Yu C. (2024). One-Step Hydrothermal Synthesis of CuS/MoS_2_ Composite for Use as an Electrochemical Non-Enzymatic Glucose Sensor. Heliyon.

[127] Bano M., Naikoo G.A., BaOmar F., Rather J.A., Hassan I.U., Sheikh R.A., Kannan P., Tambuwala M.M. (2024). Revolutionizing Glucose Monitoring: Enzyme-Free 2D-MoS_2_ Nanostructures for Ultra-Sensitive Glucose Sensors with Real-Time Health-Monitoring Capabilities. ACS Omega.

[128] Shan J., Li J., Chu X., Xu M., Jin F., Wang X., Ma L., Fang X., Wei Z., Wang X. (2018). High Sensitivity Glucose Detection at Extremely Low Concentrations Using a MoS_2_-Based Field-Effect Transistor. RSC Adv..

[129] Hu J., Dai J., Huang C., Zeng X., Wei W., Wang Z., Lin P. (2023). Organic Electrochemical Transistor with MoS_2_ Nanosheets Modified Gate Electrode for Sensitive Glucose Sensing. Sensors.

[130] Borade P.A., Ali M.A., Jahan S., Sant T., Bogle K., Panat R., Jejurikar S.M. (2021). MoS_2_ Nanosheet-Modified NiO Layers on a Conducting Carbon Paper for Glucose Sensing. ACS Appl. Nano Mater..

[131] Tian H., Wang H., Wang J., Qu G., Yu X.-F., Jiang G. (2024). Understanding the Intrinsic Reactivity of Black Phosphorus. Acc. Mater. Res..

[132] Mohadesi V. (2025). Sensitivity Enhancement of Plasmonic Biosensor for Glucose Level Detection Using Black Phosphorus and Copper-Based Multilayer Design. Plasmonics.

[133] Özkahraman E.E., Eroğlu Z., Efremov V., Maryyam A., Abbasiasl T., Das R., Mirzajani H., Hanedar B.A., Beker L., Metin O. (2025). High-Performance Black Phosphorus/Graphitic Carbon Nitride Heterostructure-Based Wearable Sensor for Real-Time Sweat Glucose Monitoring. Adv. Mater. Technol..

[134] Houari F., El Barghouti M., Mir A., Akjouj A. (2024). Nanosensors Based on Bimetallic Plasmonic Layer and Black Phosphorus: Application to Urine Glucose Detection. Sensors.

[135] Rafiee E., Negahdari R. (2023). An Efficient Biosensor Based on Plasmon-Induced Transparency Effect in Plasmonic-Graphene-Black Phosphorous Absorber for Diabetes Diagnosis. Diam. Relat. Mater..

[136] Huang C.-P., Liu S.-W., Chen T.-M., Li Y.-K. (2008). A New Approach for Quantitative Determination of Glucose by Using CdSe/ZnS Quantum Dots. Sens. Actuators B Chem..

[137] Abd Rahman S., Ariffin N., Yusof N.A., Abdullah J., Mohammad F., Ahmad Zubir Z., Nik Abd Aziz N.M.A. (2017). Thiolate-Capped CdSe/ZnS Core-Shell Quantum Dots for the Sensitive Detection of Glucose. Sensors.

[138] Duong H.D., Rhee J.I. (2007). Use of CdSe/ZnS Core-Shell Quantum Dots as Energy Transfer Donors in Sensing Glucose. Talanta.

[139] Tang Y., Yang Q., Wu T., Liu L., Ding Y., Yu B. (2014). Fluorescence Enhancement of Cadmium Selenide Quantum Dots Assembled on Silver Nanoparticles and Its Application to Glucose Detection. Langmuir.

[140] Ağbulut M.S.B., Elibol E., Çadırcı M., Demirci T. (2025). Fluorescent CdTe/ZnS Core/Shell Quantum Dots for Sensitive Metabolite Detection in Real Samples. J. Fluoresc..

[141] Wu W., Zhou T., Shena J., Zhou S. (2009). Optical Detection of Glucose by CdS Quantum Dots Immobilized in Smart Microgels. Chem. Commun..

[142] Rahmania F.J., Imae T., Chu J.P. (2024). Electrochemical Nonenzymatic Glucose Sensors Catalyzed by Au Nanoclusters on Metallic Nanotube Arrays and Polypyrrole Nanowires. J. Colloid Interface Sci..

[143] Lee M.-J., Choi J.-H., Shin J.-H., Yun J., Kim T., Kim Y.-J., Oh B.-K. (2023). Gold Nanoclusters with Two Sets of Embedded Enzyme Nanoparticles for Applications as Electrochemical Sensors for Glucose. ACS Appl. Nano Mater..

[144] Hussain A.M.P., Sarangi S.N., Kesarwani J.A., Sahu S.N. (2011). Au-Nanocluster Emission-Based Glucose Sensing. Biosens. Bioelectron..

[145] German N., Popov A., Ramanavicius A., Ramanaviciene A. (2025). A Platform for the Glucose Biosensor Based on Dendritic Gold Nanostructures and Polyaniline-Gold Nanoparticles Nanocomposite. Biosensors.

[146] Wei Y., Li Y., Liu X., Xian Y., Shi G., Jin L. (2010). ZnO Nanorods/Au Hybrid Nanocomposites for Glucose Biosensor. Biosens. Bioelectron..

[147] Wang M., Ma J., Chang Q., Fan X., Zhang G., Zhang F., Peng W., Li Y. (2018). Fabrication of a Novel ZnO–CoO/rGO Nanocomposite for Nonenzymatic Detection of Glucose and Hydrogen Peroxide. Ceram. Int..

[148] Choi Y.-B., Kim H.-S., Jeon W.-Y., Lee B.-H., Shin U.S., Kim H.-H. (2019). The Electrochemical Glucose Sensing Based on the Chitosan-Carbon Nanotube Hybrid. Biochem. Eng. J..

[149] Kang X., Mai Z., Zou X., Cai P., Mo J. (2008). Glucose Biosensors Based on Platinum Nanoparticles-Deposited Carbon Nanotubes in Sol–Gel Chitosan/Silica Hybrid. Talanta.

[150] Paik E.-S., Kim Y.-R., Hong H.-G. (2018). Amperometric Glucose Biosensor Utilizing Zinc Oxide-Chitosan-Glucose Oxidase Hybrid Composite Films on Electrodeposited Pt-Fe(III). Anal. Sci..

[151] Zalke J.B., Narkhede N.P., Rotake D.R., Singh S.G. (2024). Facile Chemiresistive Biosensor Functionalized with PANI/GOx and Novel Green Synthesized Silver Nanoparticles for Glucose Sensing. Microchem. J..

[152] Khachornsakkul K., Rybicki F.J., Sonkusale S. (2023). Nanomaterials Integrated with Microfluidic Paper-Based Analytical Devices for Enzyme-Free Glucose Quantification. Talanta.

[153] Li X., Zhao C., Liu X. (2015). A Paper-Based Microfluidic Biosensor Integrating Zinc Oxide Nanowires for Electrochemical Glucose Detection. Microsyst. Nanoeng..

[154] Chan P.Z., Jin E., Jansson M., Chew H.S.J. (2024). AI-Based Noninvasive Blood Glucose Monitoring: Scoping Review. J. Med. Internet Res..

[155] Sunstrum F.N., Khan J.U., Li N.-W., Welsh A.W. (2025). Wearable textile sensors for continuous glucose monitoring. Biosens. Bioelectron..

[156] Fu W., Yu B., Ji D., Zhou Z., Li X., Wang R., Lu W., Sun Y., Dai Y. (2024). Intelligent fibers and textiles for wearable biosensors. Responsive Mater..

[157] Moshfiq-Us-Saleheen C., Sutirtha R., Krishna Prasad A., Henry L., Richa P. (2024). Realizing the Potential of Commercial E-Textiles for Wearable Glucose Biosensing Application. ACS Mater. Au.

[158] Hosain M.N., Kwak Y.-S., Lee J., Choi H., Park J., Kim J. (2024). IoT-enabled biosensors for real-time monitoring and early detection of chronic diseases. Phys. Act. Nutr..

[159] Kharb S. (2025). Future directions: What LIES ahead for smart biochemical wearables in health monitoring?. Front. Anal. Sci..

